# Monocyte to High-Density Lipoprotein Cholesterol Ratio at the Nexus of Type 2 Diabetes Mellitus Patients With Metabolic-Associated Fatty Liver Disease

**DOI:** 10.3389/fphys.2021.762242

**Published:** 2021-12-17

**Authors:** Jue Jia, Ruoshuang Liu, Weiping Wei, Fan Yu, Xiawen Yu, Yirong Shen, Caiqin Chen, Zhensheng Cai, Chenxi Wang, Zhicong Zhao, Dong Wang, Ling Yang, Guoyue Yuan

**Affiliations:** ^1^Department of Endocrinology and Metabolism, The Affiliated Hospital of Jiangsu University, Zhenjiang, China; ^2^Department of Endocrinology and Metabolism, Jurong Hospital Affiliated to Jiangsu University, Zhenjiang, China

**Keywords:** monocyte to high-density lipoprotein cholesterol ratio (MHR), metabolic-associated fatty liver disease, type 2 diabetes mellitus, inflammatory marker, obesity

## Abstract

**Background:** Recently, monocyte to high-density lipoprotein cholesterol ratio (MHR) as a novel inflammatory biomarker has drawn lots of attention. This study was conducted in patients with type 2 diabetes mellitus (T2DM) to investigate the correlation between MHR and metabolic-associated fatty liver disease (MAFLD).

**Methods:** Totally, 1,051 patients with T2DM from the Affiliated Hospital of Jiangsu University were enrolled and classified as MAFLD (*n* = 745) group and non-MAFLD (*n* = 306) group according to the MAFLD diagnostic criteria. In contrast, patients were also separated into four groups based on MHR quartiles. Anthropometric and biochemical measurements were performed. The visceral fat area (VFA) and subcutaneous fat area (SFA) of participants were measured by dual bioelectrical impedance. Fatty liver was assessed by ultrasonography.

**Results:** The MHR level of subjects in the MAFLD group was statistically greater than that in the non-MAFLD group (*P* < 0.05). Meanwhile, MHR was higher in the overweight or obese MAFLD group compared with that in the lean MAFLD group (*P* < 0.05). The area under the ROC Curve (AUC) assessed by MHR was larger than that of other inflammatory markers (*P* < 0.01). The cutoff value of MHR was 0.388, with a sensitivity of 61.74% and a specificity of 56.54%. For further study, binary logistic regression analyses of MAFLD as a dependent variable, the relationship between MHR and MAFLD was significant (*P* < 0.01). After adjusting for many factors, the relationship still existed. In the four groups based on MHR quartiles, groups with higher values of MHR had a significantly higher prevalence of MAFLD (*P* < 0.05). The percentage of patients with obese MAFLD increased as the MHR level increased (*P* < 0.01). Among different quartiles of MHR, it showed that with the increasing of MHR, the percentage of patients with MAFLD who had more than four metabolic dysfunction indicators increased, which was 46.39, 60.52, 66.79, and 79.91%, respectively, in each quartile.

**Conclusion:** Monocyte to high-density lipoprotein cholesterol ratio is a simple and practicable inflammatory parameter that could be used for assessing MAFLD in T2DM. T2DM patients with higher MHR have more possibility to be diagnosed as MAFLD. Therefore, more attention should be given to the indicator in the examination of T2DM.

## Introduction

Currently, owing to the rapidly growing economy and unhealthy lifestyles, non-alcoholic fatty liver disease (NAFLD) has become an epidemic globally (Loomba et al., 2021; Powell et al., 2021). Its prevalence is up to ~25% (Zhou et al., 2021). It is characterized by hepatic triglyceride (TG) accumulation, and depending on the progress of the disease process, it ranges from liver steatosis to non-alcoholic steatohepatitis (NASH), fibrosis, cirrhosis, till hepatocellular carcinoma, which has taken the serious economic burden to the society (Kumar et al., 2021; Yki-Järvinen et al., 2021). Although the pathogenesis of NAFLD has not been fully clarified, previous studies showed that it shared common pathophysiological mechanisms with type 2 diabetes mellitus (T2DM), such as insulin resistance (IR), impaired lipid metabolism, and inflammation (Ferguson and Finck, 2021; Targher et al., 2021). There are also data showing that the prevalence of NAFLD in subjects with T2DM/glucose intolerance was estimated to be higher (around 40–70%) than that in the general population (Younossi et al., 2019; Mantovani et al., 2020). Meanwhile, NAFLD is often accompanied by serious complications, for instance, cardiovascular diseases and chronic kidney diseases, thus leading to a bad prognosis for patients with T2DM (Mantovani et al., 2020; Nasr et al., 2020). In 2020, NAFLD was renamed metabolic-associated fatty liver disease (MAFLD), which is a sensitive and important indicator of metabolic dysfunction (Eslam et al., 2020).

In recent years, studies indicate that inflammation plays an important role in the pathophysiology of NAFLD (Han et al., 2021; Zhang et al., 2021). Lipotoxicity and release of endogenous factors induce the hepatic inflammatory response (Han et al., 2021). Inflammatory cells, such as neutrophils, lymphocytes, monocytes, macrophages, and Kupffer cells, infiltrated in the liver could mediate hepatic TG storage, regulate the inflammatory response, lead to the phenomena of lipid peroxidation, produce their own reactive oxygen species, and activate nuclear transcription factors, which contribute to hepatocellular damage (Wang et al., 2020; Sakurai et al., 2021; Tacke and Weiskirchen, 2021).

Recently, monocyte to high-density lipoprotein cholesterol ratio (MHR) as a novel inflammatory biomarker, which is largely available in clinical practice, has drawn lots of attention. Elevated MHR has been proved to be associated with many disorders such as cardiovascular diseases, metabolic syndrome (MetS), and polycystic ovary syndrome (PCOS) (Akboga et al., 2016; Uslu et al., 2018; Usta et al., 2018). Studies also investigated that increased MHR was independently related to long-term mortality in patients with coronary artery disease (CAD) who have undergone percutaneous coronary intervention (Zhang et al., 2020). MHR is also a marker that could predict the presence and progression of subclinical carotid atherosclerosis in patients with T2DM (Chen et al., 2019). However, till now, no data exist regarding the association between MHR and T2DM patients with MAFLD. In this study, we aimed to evaluate the association between the inflammatory biomarker MHR and T2DM patients with MAFLD.

## Methods

### Study Population

**Figure 1:**
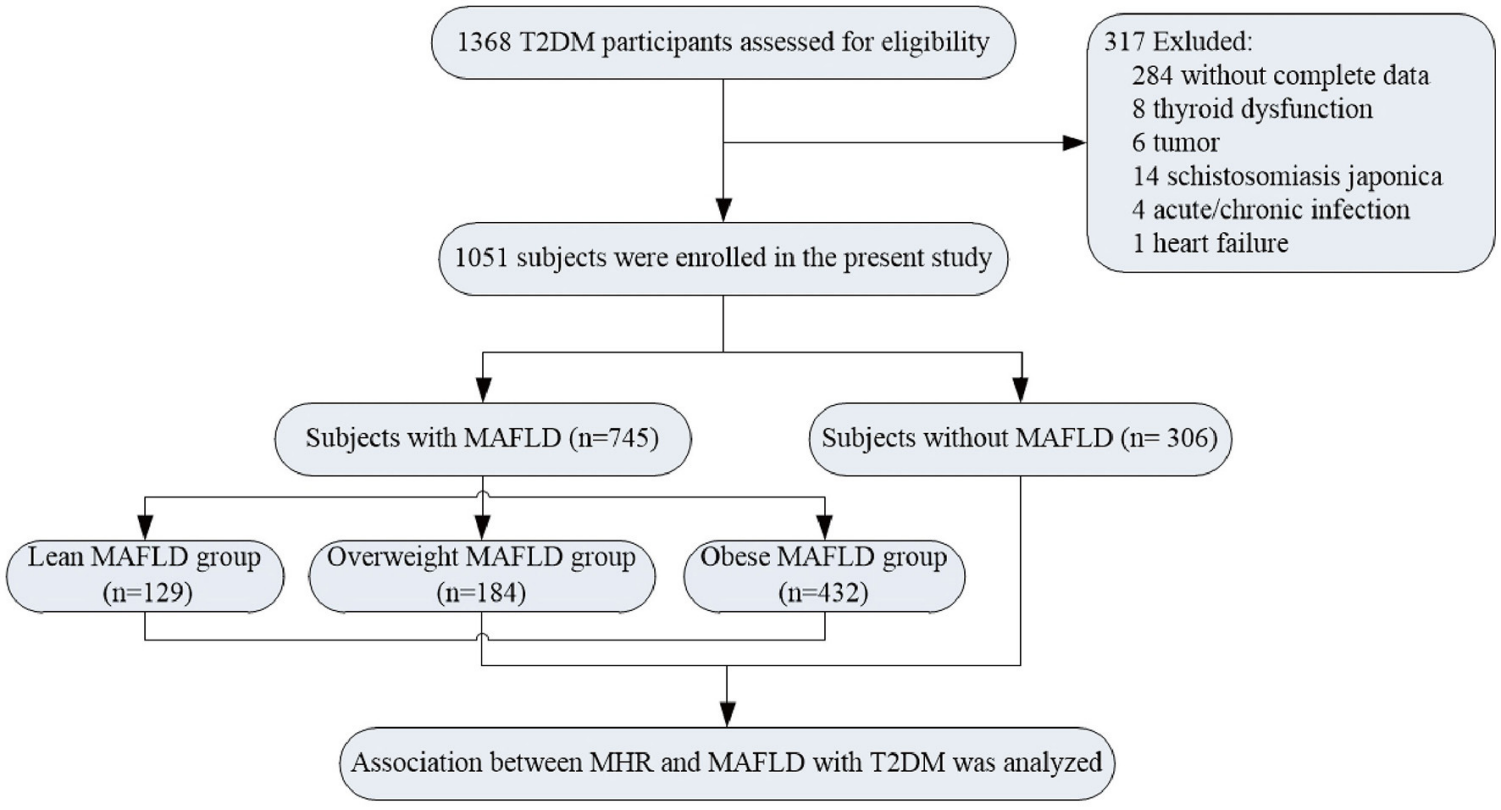
Flowchart describing the selection process of the study population.

This study upholds the principles of the Declaration of Helsinki, and the study protocol was approved by the Human Research Ethics Committee of the Affiliated Hospital of Jiangsu University. All the patients recruited in this study signed informed consent. A total of 1,368 participants with T2DM were enrolled in the study population from June 2018 to July 2020. T2DM was diagnosed according to the criteria of the American Diabetes Association (American Diabetes Association, 2021). According to the diagnostic criteria of MAFLD (Eslam et al., 2020), the patients were separated into two groups, namely, non-MAFLD group and MAFLD group. The exclusion criteria were as follows: type 1 diabetes, gestational diabetes mellitus, special type diabetes, acute/chronic infection, autoimmune disease, hematological disease, chronic lung disease, tumor, thyroid dysfunction, and those without complete data. Thus, 317 individuals were excluded from this study. Eventually, 1,051 patients were included in the final enrollment (Figure 1). In the meantime, on the basis of body mass index (BMI) values (Shin and Lee, 2021), the MAFLD group was separated into lean MAFLD group (BMI < 23 kg/m^2^), overweight MAFLD group (BMI, 23.0–24.9 kg/m^2^), and obese MAFLD group (BMI ≥ 25.0 kg/m^2^). The criteria of metabolic dysfunction were as follows (Lima et al., 2015; Osonoi et al., 2018; Blanquet et al., 2019): (1) waist circumference (WC) ≥ 90 cm for men and ≥ 80 cm for women; (2) systolic blood pressure (SBP) ≥ 130 mmHg or diastolic blood pressure (DBP) ≥ 85 mmHg or treatment of previously diagnosed hypertension; (3) TG levels ≥ 1.70 mmol/L or specific treatment for this lipid abnormalities; (4) high-density lipoprotein cholesterol levels (HDL-c) of < 1.0 mmol/L in men and < 1.3 mmol/L in women or specific treatment for this lipid abnormalities; (5) fasting plasma glucose of ≥5.60 mmol/L or previously diagnosed T2DM; (6) uric acid levels of ≥420 μmol/L or specific treatment for this abnormalities; and (7) urinary microalbumin (uMA) > 30 mg/L or MA/UCREA > 30 mg/L.

### Clinical and Biochemical Parameters

Anthropometric indexes of patients such as height, weight, BMI, neck circumference (NC), WC, hip waist circumference (HC), blood pressure (BP), and heart rate were measured by trained survey personnel in accordance with international standards. After overnight fasting for longer than 8 h, the venous blood samples of subjects were collected. The glucose oxidase method was used to detect fasting blood glucose, and the chemiluminescence method was used to determine fasting plasma insulin and C-peptide. HbA1c was measured by high-performance liquid chromatography (HPLC). Serum lipids and liver function indicators were detected using a BEKMAN AU 5800 automatic biochemical analyzer. Monocyte, neutrophil, and lymphocyte counts were determined using an SYSMEX XN3000 automated blood cell counter.

Homeostasis model assessment was used to estimate insulin resistance (HOMA-IR). HOMA-IR = fasting plasma glucose (mmol/L) × fasting plasma insulin (mIU/L)/22.5. Insulin sensitivity index (ISI) was conducted to estimate insulin sensitivity. ISI = 22.5/fasting plasma glucose (mmol/L) × fasting plasma insulin (mIU/L).

### Measurement of Visceral Fat Area

The visceral fat area (VFA) and subcutaneous fat area (SFA) of participants were measured using a dual bioelectrical impedance at the umbilical level (DUALSCAN; OmronHealthcare Co. Ltd, Kyoto, Japan).

### Calculation of MHR and Other Inflammatory Markers

The MHR, neutrophil to HDL-c ratio (NHR), neutrophil to lymphocyte ratio (NLR), and platelet to lymphocyte ratio (PLR) were calculated using the following formula, respectively: MHR = monocyte/HDL-c, NHR = neutrophil/HDL-c, NLR = neutrophil/lymphocyte, and PLR = platelet/lymphocyte.

### Assessment of Fatty Liver by Ultrasonography

Liver ultrasonography was performed by experienced sonographers. All the patients fasted overnight for 8 h before ultrasound imaging. The diagnostic criteria of hepatic steatosis were based on the following sonographic characteristics: enlarged or slightly normal liver volume, full in shape, and obtuse at both lower margins; increased liver contrast compared with kidney and spleen; flake hypoechoic areas can be seen in some parenchyma; intrahepatic biliary tract is not clearly shown; and the echo of portal vein wall is weakened.

### Calculation of the NAFLD Fibrosis Score

The non-alcoholic fatty liver disease fibrosis score (NFS) was used to evaluate the degree of liver fibrosis, and the calculation formula was as follows: NFS = −1.675 + 0.037 × age (years) + 0.094 × BMI (kg/m^2^) + 1.13 × impaired fasting glucose/diabetes (yes = 1, no = 0) + 0.99 × AST/ALT ratio − 0.013 × platelet count (×10^9^/L) − 0.66 × albumin (g/dl) (Bril et al., 2020).

Advanced fibrosis was explicitly excluded if the NFS was lower than the cutoff point (−1.455), while the diagnosis of advanced fibrosis was established when the NFS was above the cutoff point (0.675) (Bril et al., 2020).

### Statistical Analysis

Statistical analyses were performed using SPSS version 22.0 software (SPSS, Inc., Chicago, IL, United States). Continuous variables were described as mean values ± SD or median (interquartile range) according to the distributions of data. Categorical variables were expressed as the number of patients and percentage. The difference between the two groups was examined using Student's *t*-test or Mann-Whitney *U* test, and the difference among the three groups was determined using the one-way ANOVA (normally distributed variables) or Kruskal-Wallis test (non-normally distributed variables). The chi-squared test was used for categorical variables. The relationship between variables was tested by the Pearson or Spearman correlation analysis. Binary logistics regression analyses were performed to explore the association of MHR with MAFLD. The receiver operating characteristic (ROC) curves were operated to identify the optimal value for the assessment of the risk of MAFLD in this population. Optimal cutoffs were derived from maximizing the Yoden index. A *p* < 0.05 (two-sided) was defined as statistically significant.

## Results

### The Clinical, Biochemical, and Inflammatory Characteristics of Patients

In this current cohort of 1,051 patients (Supplementary Table 1), 745 subjects were MAFLD with T2DM, and the prevalence of MAFLD in T2DM was 70.88%. Male patients in both groups were more than 60%. The general characteristics of participants were presented in Table 1. With regard to the demographic parameters, patients with MAFLD were younger, showing a higher percentage of hypertension as well as dyslipidemia than those without MAFLD (*P* < 0.05). Regarding anthropometric parameters, the MAFLD group had a remarkably higher level of height, weight, BMI, NC, WC, HC, DBP, MAP, VFA, and SFA than the non-MAFLD group. Regarding biochemical parameters, fasting plasma insulin, fasting C-peptide, 2-h plasma insulin, 2-h C-peptide, HOMA-IR, ALT, AST, γ-glutamyl transpeptidase (γ-GGT), albumin, uric acid, total cholesterol (TCHOL), and TG were significantly augmented in patients with MAFLD compared with those with non-MAFLD, while HbA1c, HOMA-ISI, urea nitrogen, and HDL-c were greatly reduced in subjects with MAFLD (*P* < 0.05). Concerning immune cell counts, lymphocyte counts, MHR, and NHR levels were statistically greater in the MAFLD group than the non-MAFLD group (*P* < 0.05).

**Table 1:** The clinical, biochemical, and inflammatory characteristics of patients in MAFLD and non-MAFLD groups.

**Variables**	**MAFLD (*n* = 745)**	**Non-MAFLD (*n* = 306)**	***P* value**
**Demographic parameters**			
Age (years)	**55.00 (47.00, 69.00)**	**60.00 (52.00, 66.00)**	**<0.001**
Male (*n*, %)	454 (60.94)	189 (61.76)	0.803
Smoking (*n*, %)	201 (26.98)	77 (25.16)	0.544
Alcohol intake (*n*, %)	103 (13.83)	36 (11.76)	0.370
Hypertension (*n*, %)	**434 (58.26)**	**137 (44.77)**	**<0.001**
History of CAD (*n*, %)	59 (7.92)	23 (7.52)	0.825
Dyslipidemia (*n*, %)	**663 (88.99)**	**179 (58.50)**	**<0.001**
Antidiabetic drug (*n*, %)	**587 (78.79)**	**265 (86.60)**	**0.003**
**Anthropometric parameters**			
Height (cm)	**167.00 (160.00, 173.00)**	**165.00 (159.50, 170.50)**	**0.001**
Weight (kg)	**70.70 (63.60, 79.25)**	**62.60 (56.68, 68.3)**	**<0.001**
BMI (kg/m^2^)	**25.60 (23.70, 27.73)**	**23.00 (21.40, 25.00)**	**<0.001**
NC (cm)	**39.27 ± 4.75**	**37.00 ± 4.39**	**<0.001**
WC (cm)	**93.67 ± 8.98**	**85.85 ± 8.99**	**<0.001**
HC (cm)	**98.60 ± 7.85**	**94.63 ± 7.17**	**<0.001**
SBP (mmHg)	129.24 ± 16.77	127.06 ± 17.91	0.061
DBP (mmHg)	**76.01 ± 10.29**	**71.84 ± 9.82**	**<0.001**
MAP (mmHg)	**93.75 ± 11.14**	**90.24 ± 10.91**	**<0.001**
VFA (cm^2^)	**102.98 ± 34.47**	**66.22 ± 33.56**	**<0.001**
SFA (cm^2^)	**190.00 (154.40, 229.75)**	**142.65 (116.00, 177.63)**	**<0.001**
**Biochemical parameters**			
Fasting plasma glucose (mmol/L)	9.84 (7.87, 12.54)	9.97 (7.24, 12.90)	0.846
Fasting plasma insulin (μIU/mL)	**8.22 (5.08, 11.19)**	**5.38 (3.17, 10.30)**	**<0.001**
Fasting C-peptide (ng/mL)	**2.68 ± 1.01**	**1.82 ± 1.02**	**<0.001**
2 h plasma glucose (mmol/L)	19.16 ± 5.10	19.25 ± 5.51	0.792
2 h plasma insulin (μIU/mL)	**32.87 (18.12, 45.47)**	**26.97 (13.14, 39.17)**	**<0.001**
2 h C-peptide (ng/mL)	**4.91 (3.66, 7.39)**	**3.51 (2.33, 5.02)**	**<0.001**
HbA1c (%)	**9.40 (8.00, 10.80)**	**9.70 (7.98, 11.50)**	**0.027**
HOMA-IR	**3.68 (2.27, 4.92)**	**2.34 (1.41, 3.74)**	**<0.001**
HOMA-ISI	**0.32 (0.20, 0.44)**	**0.51 (0.29, 0.71)**	**<0.001**
ALT (U/L)	**24.00 (16.20, 40.50)**	**16.60 (11.00, 23.25)**	**<0.001**
AST (U/L)	**18.60 (14.20, 26.00)**	**15.20 (12.30, 20.00)**	**<0.001**
ALP (U/L)	71.00 (58.00, 87.00)	71.40 (58.00, 84.00)	0.728
γ-GGT (U/L)	**33.00 (23.00, 52.00)**	**22.00 (15.75, 32.00)**	**<0.001**
Albumin (g/L)	**40.70 (38.70, 42.70)**	**39.40 (37.20, 42.00)**	**<0.001**
Blood urea nitrogen (mmol/L)	**5.13 (4.27, 6.25)**	**5.51 (4.60, 6.87)**	**<0.001**
Creatinine (μmol/L)	59.00 (49.80, 69.50)	60.45 (49.10, 70.80)	0.333
Uric acid (μmol/L)	**298.00 (243.00, 351.50)**	**258.00 (208.50, 310.25)**	**<0.001**
TCHOL (mmol/L)	**4.96 ± 1.14**	**4.77 ± 1.24**	**0.021**
TG (mmol/L)	**2.23 (1.56, 3.25)**	**1.44 (1.00, 1.99)**	**<0.001**
HDL-c (mmol/L)	**1.02 (0.86, 1.21)**	**1.15 (0.97, 1.45)**	**<0.001**
LDL-c (mmol/L)	2.83 ± 0.90	2.79 ± 0.98	0.515
Blood Cells Counts			
WBC (*10^9^/L)	6.00 (5.00, 7.10)	5.80 (4.80, 7.20)	0.102
Neutrophil (*10^9^/L)	3.30 (2.70, 4.20)	3.30 (2.50, 4.40)	0.909
Monocyte (*10^9^/L)	0.47 ± 0.15	0.46 ± 0.16	0.298
Lymphocyte (*10^9^/L)	**2.00 (1.60, 2.40)**	**1.80 (1.40, 2.20)**	**<0.001**
Platelet (*10^9^/L)	195.26 ± 55.78	190.84 ± 54.47	0.240
**Inflammation parameters**			
MHR	**0.43 (0.33, 0.58)**	**0.37 (0.27, 0.49)**	**<0.001**
NHR	**3.24 (2.44, 4.29)**	**2.90 (1.99, 4.17)**	**<0.001**
NLR	**1.67 (1.28, 2.20)**	**1.79 (1.40, 2.67)**	**0.002**
PLR	**96.67 (75.29, 122.05)**	**103.10 (79.37, 135.88)**	**0.004**

*CAD, coronary artery disease; BMI, body mass index; NC, neck circumference; WC, waist circumference; HC, hip circumference; SBP, systolic blood pressure; DBP, diastolic blood pressure; MAP, mean arterial pressure; VFA, visceral fat area; SFA, subcutaneous fat area; HOMA-IR, homeostasis model assessment of insulin resistance; HOMA-ISI, homeostasis model assessment of insulin sensitivity index; ALT, alanine aminotransferase; AST, aspartate aminotransferase; ALP, alkaline phosphatase; γ-GGT, gamma-glutamyl transferase; TCHOL, total cholesterol; TG, triglyceride; HDL-c, high-density lipoprotein cholesterol; LDL-c, low-density lipoprotein cholesterol; WBC, white blood cell; MHR, monocyte/HDL-c; NHR, neutrophil/HDL-c; NLR, neutrophil/lymphocyte; PLR, platelet/lymphocyte. Continuous variables were described as mean values ± SD and median (interquartile range) according to the distributions of data. Categorical variables were expressed as the number of patients and percentage. P <0.05 (two-sided) was defined as statistically significant. Bold values indicate statistically significance*.

### Subgroup Analysis of the Clinical and Laboratory Characteristics Based on BMI in Patients With MAFLD

As shown in Table 2, the parameters of weight, NC, WC, HC, SBP, DBP, MAP, VFA, SFA, fasting plasma insulin, fasting C-peptide, 2-h plasma insulin, HOMA-IR, HOMA-ISI, ALT, AST, γ-GGT, uric acid, TG, and HDL-c presented a remarkable difference among lean MAFLD group, overweight MAFLD group, and obese MAFLD group (*P* < 0.05). The inflammation markers of MHR and NHR were higher in the overweight or obese MAFLD group compared with those in the lean MAFLD group (*P* < 0.05). NFS of the highest BMI group increased than that of lower BMI groups (*P* < 0.05), while no difference in fibrosis severity scale was observed in different BMI groups.

**Table 2:** Subgroup analysis of the clinical and laboratory characteristics based on BMI in patients with MAFLD.

**Variables**	**Lean MAFLD (*n* = 129)**	**Overweight MAFLD (*n* = 184)**	**Obese MAFLD (*n* = 432)**	***P* Value**
	**(BMI <23 kg/m^**2**^)**	**(BMI 23.0–24.9 kg/m^**2**^)**	**(BMI ≥ 25.0 kg/m2)**	
Age (years)	56.00 (50.00, 63.00)	56.00 (48.00, 63.00)	55.00 (45.00, 64.00)	0.279
Male (*n*, %)	76 (58.91)	112 (60.87)	266 (61.57)	0.863
Smoking (*n*, %)	30 (23.26)	57 (30.98)	114 (26.39)	0.290
Alcohol intake (*n*, %)	20 (15.50)	28 (15.22)	55 (12.73)	0.595
Hypertension (*n*, %)	**58 (44.96)**	**94 (51.09)**	**282 (65.28)**	**<0.001**
History of CAD (*n*, %)	13 (10.08)	12 (6.52)	34 (7.87)	0.517
Dyslipidemia (*n*, %)	114 (88.37)	160 (86.96)	389 (90.05)	0.517
Antidiabetic drug (*n*, %)	108 (83.72)	144 (78.26)	335 (77.55)	0.315
Height (cm)	167.50 (160.50, 174.25)	166.00 (158.50, 173.00)	167.25 (160.00, 173.00)	0.681
Weight (kg)	**60.80 (55.75, 66.50)**	**66.25 (60.48, 72.35)**	**76.95 (69.83, 84.45)**	**<0.001**
NC (cm)	**36.00 (34.00, 39.00)**	**38.00 (36.00, 40.00)**	**40.75 (38.00, 43.00)**	**<0.001**
WC (cm)	**85.00 (80.00, 89.00)**	**89.00 (85.00, 93.00)**	**97.00 (93.00, 103.00)**	**<0.001**
HC (cm)	**93.00 (89.00, 95.00)**	**96.00 (93.00, 98.00)**	**101.00 (98.00, 106.00)**	**<0.001**
SBP (mmHg)	**124.00 (116.00, 135.00)**	**125.00 (115.00, 137.00)**	**130.00 (118.00, 141.75)**	**0.005**
DBP (mmHg)	**74.27 ± 9.60**	**75.14 ± 9.53**	**76.89 ± 10.71**	**0.016**
MAP (mmHg)	**91.46 ± 10.27**	**92.62 ± 10.75**	**94.91 ± 11.42**	**0.002**
VFA (cm^2^)	**76.62 ± 25.26**	**86.78 ± 23.50**	**117.75 ± 33.00**	**<0.001**
SFA (cm^2^)	**137.10 (116.20, 160.45)**	**162.05 (142.03, 187.60)**	**218.95 (189.00, 260.75)**	**<0.001**
Fasting plasma glucose (mmol/L)	9.07 (7.68, 11.64)	10.16 (7.80, 12.70)	9.90 (7.95, 12.81)	0.140
Fasting plasma insulin (μIU/mL)	**6.59 (4.15, 10.66)**	**6.42 (4.16, 10.09)**	**9.85 (6.17, 12.86)**	**<0.001**
Fasting C-peptide (ng/mL)	**2.40 ± 0.90**	**2.55 ± 0.89**	**2.83 ± 1.07**	**<0.001**
2h plasma glucose (mmol/L)	18.94 ± 5.16	19.44 ± 5.51	19.11 ± 4.90	0.208
2h plasma insulin (μIU/mL)	**30.01 (15.42, 39.59)**	**26.46 (15.77, 40.22)**	**37.78 (20.97, 50.37)**	**<0.001**
2h C-peptide (ng/mL)	4.68 (3.65, 7.22)	4.86 (3.64, 6.62)	5.09 (3.70, 7.54)	0.212
HbA1c (%)	9.50 (8.00, 10.80)	9.60 (8.00, 10.90)	9.20 (7.90, 10.80)	0.646
HOMA-IR	**2.71 (1.70, 4.60)**	**3.00 (1.74, 4.66)**	**4.22 (2.71, 5.76)**	**<0.001**
HOMA-ISI	**0.38 (0.28, 0.59)**	**0.37 (0.23, 0.58)**	**0.27 (0.17, 0.38)**	**<0.001**
ALT (U/L)	**18.80 (13.65, 29.25)**	**22.00 (15.48, 34.45)**	**28.60 (18.55, 45.00)**	**<0.001**
AST (U/L)	**17.10 (13.20, 21.30)**	**17.00 (13.23, 24.48)**	**20.35 (15.60, 27.70)**	**<0.001**
ALP (U/L)	70.00 (55.50, 85.00)	72.00 (57.00, 89.75)	70.50 (58.00, 86.75)	0.628
γ-GGT (U/L)	**27.00 (21.00, 46.54)**	**30.00 (22.00, 44.75)**	**35.00 (24.00, 57.00)**	**0.002**
Albumin (g/L)	40.49 ± 3.51	40.84 ± 4.52	40.80 ± 3.69	0.685
Blood urea nitrogen (mmol/L)	5.24 (4.27, 6.22)	5.07 (4.10, 6.06)	5.15 (4.31, 6.41)	0.461
Creatinine (μmol/L)	58.40 (50.1, 66.15)	59.70 (49.35, 69.68)	59.00 (50.00, 70.25)	0.628
Uric acid (μmol/L)	**286.76 ± 74.89**	**293.86 ± 75.77**	**315.86 ± 92.99**	**0.001**
TCHOL (mmol/L)	4.83 (4.19, 5.75)	4.76 (4.09, 5.60)	4.87 (4.21, 5.61)	0.462
TG (mmol/L)	**2.08 (1.39, 3.18cc)**	**2.11 (1.51, 3.12)**	**2.30 (1.63, 3.33)**	**0.029**
HDL-c (mmol/L)	**1.09 (0.95, 1.28)**	**1.02 (0.86, 1.18)**	**1.00 (0.84, 1.19)**	**0.003**
LDL-c (mmol/L)	2.87 ± 0.95	2.79 ± 0.85	2.83 ± 0.91	0.718
WBC (*10^9^/L)	5.70 (4.80, 7.00)	5.90 (4.93, 6.80)	6.20 (5.10, 7.28)	0.119
Neutrophil (*10^9^/L)	3.20 (2.60, 4.25)	3.10 (2.60, 3.90)	3.40 (2.70, 4.20)	0.119
Monocyte (*10^9^/L)	0.40 (0.40, 0.50)	0.40 (0.33, 0.50)	0.50 (0.0.40, 0.60)	0.074
Lymphocyte (*10^9^/L)	1.90 (1.45, 2.30)	2.00 (1.63, 2.40)	2.00 (1.60, 2.40)	0.099
Platelet (*10^9^/L)	198.07 ± 53.61	193.93 ± 58.36	194.99 ± 55.40	0.802
MHR	**0.39 (0.31, 0.53)**	**0.40 (0.32, 0.56)**	**0.45 (0.35, 0.60)**	**0.008**
NHR	**3.02 (2.21, 4.27)**	**3.13 (2.38, 4.05)**	**3.33 (2.57, 4.35)**	**0.040**
NLR	**1.75 (1.37, 2.38)**	**1.58 (1.27, 1.96)**	**1.70 (1.24, 2.21)**	**0.025**
PLR	103.64 (78.63, 137.75)	93.33 (71.16, 119.67)	96.98 (75.25, 121.24)	0.056
NFS	**−0.73 ± 1.05**	**−0.68 ± 1.09**	**−0.37 ± 1.12**	**<0.001**
Fibrosis severity scale				
F0–F2	31 (24.03%)	39 (21.20%)	69 (15.97%)	0.071
Indeterminant score	85 (65.89%)	126 (68.48%)	297 (68.75%)	0.825
F3–F4	13 (10.08%)	19 (10.32%)	66 (15.28%)	0.131

*CAD, coronary artery disease; BMI, body mass index; NC, neck circumference; WC, waist circumference; HC, hip circumference; SBP, systolic blood pressure; DBP, diastolic blood pressure; MAP, mean arterial pressure; VFA, visceral fat area; SFA, subcutaneous fat area; HOMA-IR, homeostasis model assessment of insulin resistance; HOMA-ISI, homeostasis model assessment of insulin sensitivity index; ALT, alanine aminotransferase; AST, aspartate aminotransferase; ALP, alkaline phosphatase; γ-GGT, gamma-glutamyl transferase; TCHOL, total cholesterol; TG, triglyceride; HDL-c, high-density lipoprotein cholesterol; LDL-c, low-density lipoprotein cholesterol; WBC, white blood cell; MHR, monocyte/HDL-c; NHR, neutrophil/HDL-c; NLR, neutrophil/lymphocyte; PLR, platelet/lymphocyte; NFS, non-alcoholic fatty liver disease fibrosis score. Bold values indicate statistically significance*.

### Correlation of MHR With Other Parameters in the Whole Study Population or MAFLD With T2DM Patients

**Figure 2:**
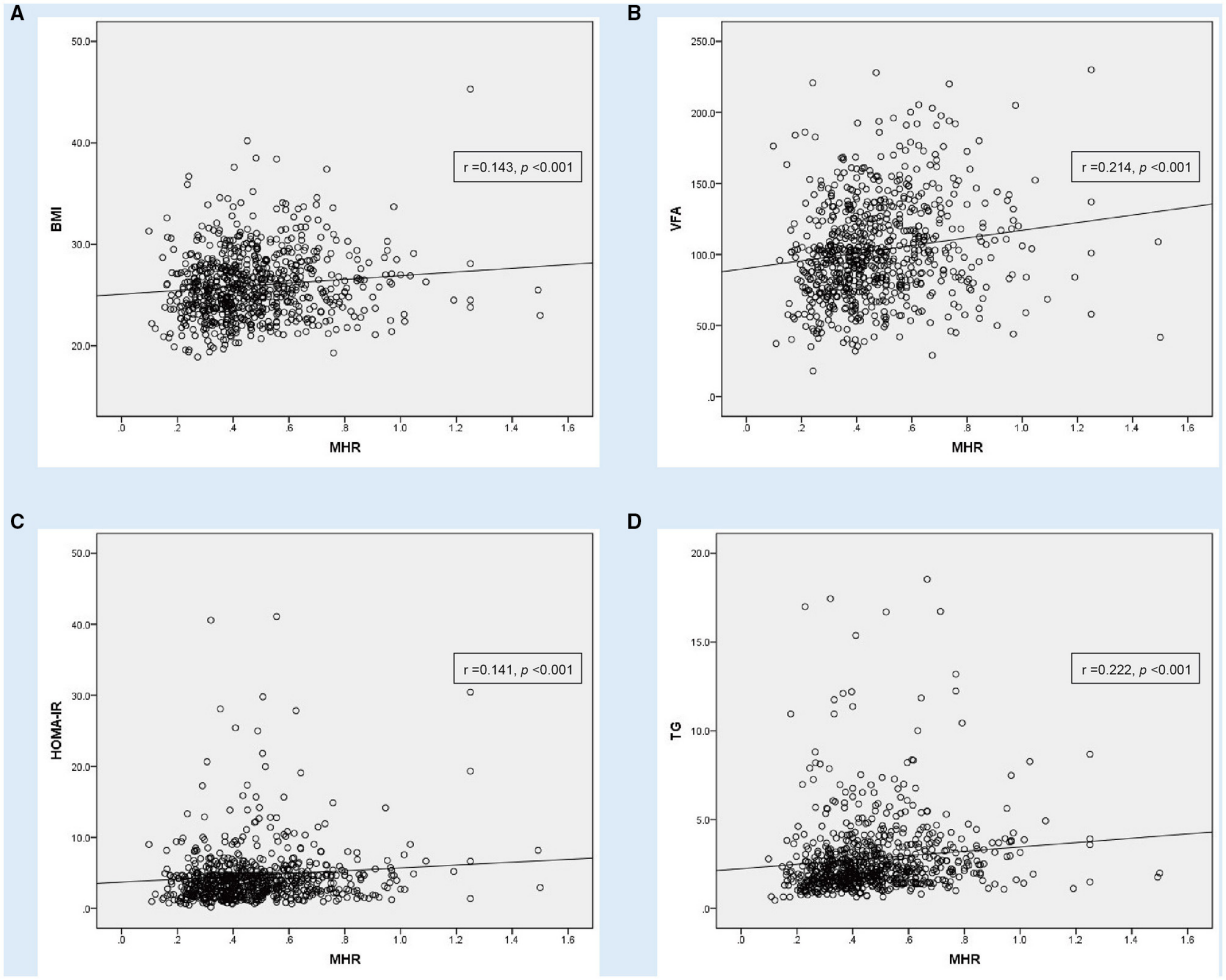
Correlation analysis showing statistically positive correlation between monocyte to HDL cholesterol ratio (MHR) with other parameters in MAFLD patients with T2DM (*n* = 745). **(A)** body mass index (BMI), **(B)** visceral fat area (VFA), **(C)** homeostasis model assessment of insulin resistance (HOMA-IR) and **(D)** triglyceride (TG).

**Figure 3:**
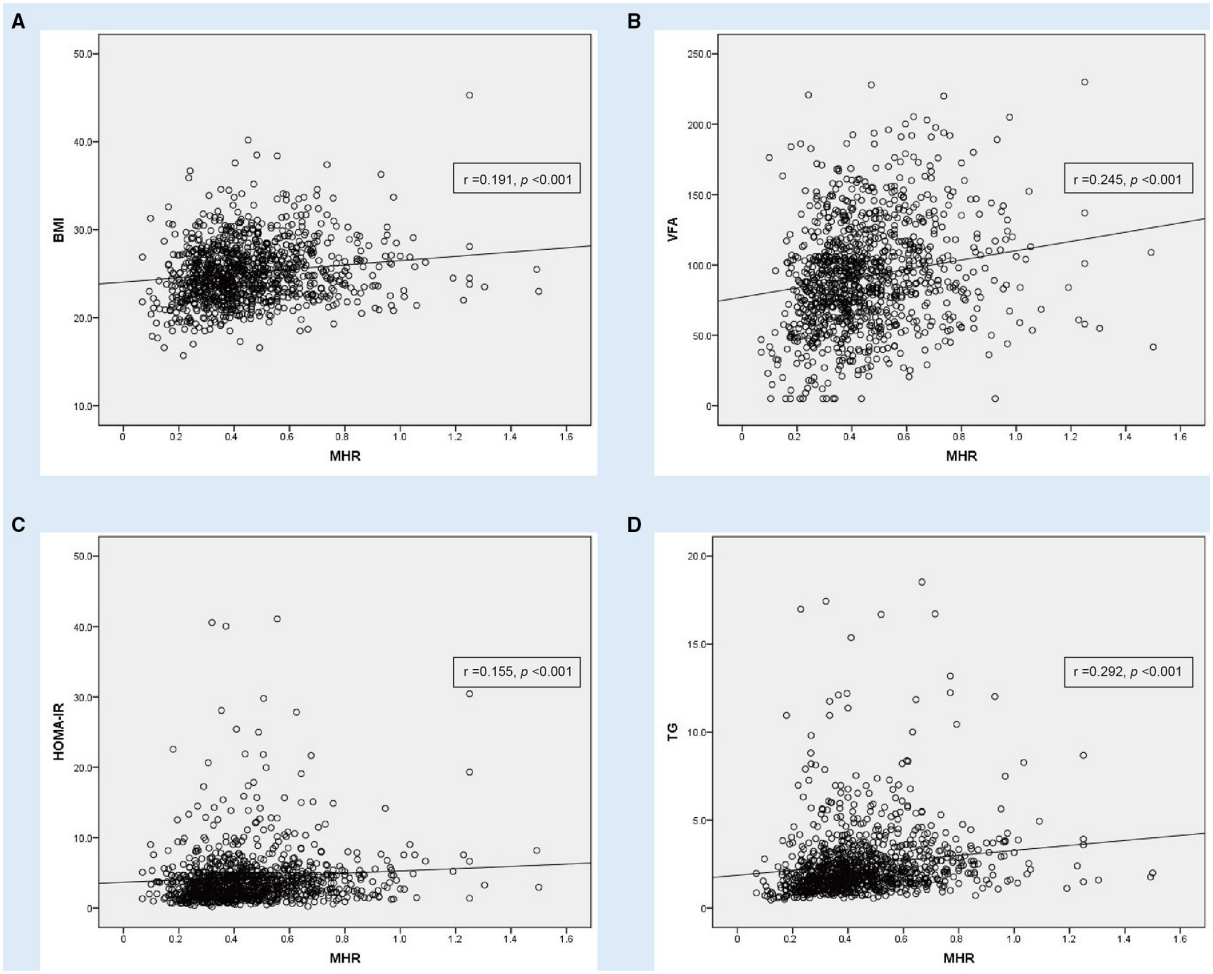
Correlation analysis showing statistically positive correlation between monocyte to HDL cholesterol ratio (MHR) with other parameters in T2DM (*n* = 1,051). **(A)** Body mass index (BMI), **(B)** visceral fat area (VFA), **(C)** homeostasis model assessment of insulin resistance (HOMA-IR) and **(D)** triglyceride (TG).

In the Pearson or Spearman correlation analysis, MHR presented a significantly positive correlation with smoking, dyslipidemia, height, weight, BMI, NC, WC, HC, VFA, fasting plasma insulin, fasting C-peptide, HOMA-IR, ALT, γ-GGT, creatinine, uric acid, TG, WBC, neutrophil, monocyte, lymphocyte, platelet, NLR, and NHR, and in the meantime, negative correlation with gender, antidiabetic drug usage, age, HOMA-ISI, TCHOL, HDL-c, LDL-c, PLR, and in both, the whole study population and MAFLD with T2DM patients (*P* < 0.05). A significantly positive correlation between MHR and other parameters, such as DBP, SFA, 2-h plasma insulin, and 2-h C-peptide, only existed in the whole population, while a positive correlation between MHR and fasting plasma glucose only existed in MAFLD with T2DM patients (*P* < 0.05). All details are shown in Table 3; Figures 2, 3.

**Table 3:** Correlation of MHR with other parameters in the whole study population or MAFLD patients with T2DM.

	**MAFLD group**		**Total**	
	**r**	***p***	**r**	***p***
Gender	**−0.344**	**<0.001**	**−0.298**	**<0.001**
Age (years)	**−0.148**	**<0.001**	**−0.159**	**<0.001**
Smoking	**0.228**	**<0.001**	**0.192**	**<0.001**
Alcohol intake	0.050	0.176	0.034	0.274
Hypertension	0.014	0.694	0.030	0.338
History of CAD	0.025	0.499	0.001	0.962
Dyslipidemia	**0.206**	**<0.001**	**0.305**	**<0.001**
Antidiabetic drug	**−0.077**	**0.035**	**−0.077**	**0.012**
Height (cm)	**0.285**	**<0.001**	**0.275**	**<0.001**
Weight (kg)	**0.292**	**<0.001**	**0.316**	**<0.001**
BMI (kg/m^2^)	**0.143**	**<0.001**	**0.191**	**<0.001**
NC (cm)	**0.310**	**<0.001**	**0.313**	**<0.001**
WC (cm)	**0.215**	**<0.001**	**0.256**	**<0.001**
HC (cm)	**0.114**	**0.002**	**0.143**	**<0.001**
SBP (mmHg)	0.029	0.428	0.021	0.498
DBP (mmHg)	0.046	0.209	**0.079**	**0.010**
MAP (mmHg)	0.040	0.281	0.060	0.053
VFA (cm^2^)	**0.214**	**<0.001**	**0.245**	**<0.001**
SFA (cm^2^)	0.059	0.110	**0.128**	**<0.001**
Fasting plasma glucose (mmol/L) (mmol/L)	**0.079**	**0.031**	0.053	0.085
Fasting plasma insulin (μIU/mL)	**0.119**	**0.001**	**0.130**	**<0.001**
Fasting C-peptide (ng/mL)	**0.126**	**0.001**	**0.205**	**<0.001**
2 h plasma glucose (mmol/L)	0.060	0.102	0.053	0.088
2 h plasma insulin (μIU/mL)	0.027	0.455	**0.067**	**0.029**
2 h C-peptide (ng/mL)	0.011	0.763	**0.102**	**0.001**
HbA1c (%)	0.059	0.106	0.036	0.242
HOMA-IR	**0.141**	**<0.001**	**0.155**	**<0.001**
HOMA-ISI	**−0.129**	**<0.001**	**−0.166**	**<0.001**
ALT (U/L)	**0.144**	**<0.001**	**0.158**	**<0.001**
AST (U/L)	0.022	0.549	0.034	0.268
ALP (U/L)	**–**0.060	0.099	**–**0.030	0.324
γ-GGT (U/L)	**0.158**	**<0.001**	**0.212**	**<0.001**
Albumin (g/L)	**–**0.057	0.119	**−0.078**	**0.011**
Blood urea nitrogen (mmol/L)	**0.085**	**0.020**	0.037	0.228
Creatinine (μmol/L)	**0.157**	**<0.001**	**0.132**	**<0.001**
Uric acid (μmol/L)	**0.199**	**<0.001**	**0.215**	**<0.001**
TCHOL (mmol/L)	**−0.177**	**<0.001**	**−0.165**	**<0.001**
TG (mmol/L)	**0.222**	**<0.001**	**0.292**	**<0.001**
HDL-c (mmol/L)	**−0.645**	**<0.001**	**−0.666**	**<0.001**
LDL-c (mmol/L)	**−0.169**	**<0.001**	**−0.123**	**<0.001**
WBC (*10^9^/L)	**0.462**	**<0.001**	**0.469**	**<0.001**
Neutrophil (*10^9^/L)	**0.365**	**<0.001**	**0.367**	**<0.001**
Monocyte(*10^9^/L)	**0.757**	**<0.001**	**0.744**	**<0.001**
Lymphocyte (*10^9^/L)	**0.264**	**<0.001**	**0.292**	**<0.001**
Platelet (*10^9^/L)	**0.159**	**<0.001**	**0.148**	**<0.001**
NLR	**0.087**	**0.018**	**0.075**	**0.016**
NHR	**0.667**	**<0.001**	**0.669**	**<0.001**
PLR	**−0.117**	**0.001**	**−0.147**	**<0.001**

*CAD, coronary artery disease; BMI, body mass index; NC, neck circumference; WC, waist circumference; HC, hip circumference; SBP, systolic blood pressure; DBP, diastolic blood pressure; MAP, mean arterial pressure; VFA, visceral fat area; SFA, subcutaneous fat area; HOMA-IR, homeostasis model assessment of insulin resistance; HOMA-ISI, homeostasis model assessment of insulin sensitivity index; ALT, alanine aminotransferase; AST, aspartate aminotransferase; ALP, alkaline phosphatase; γ-GGT, gamma-glutamyl transferase; TCHOL, total cholesterol; TG, triglyceride; HDL-c, high-density lipoprotein cholesterol; LDL-c, low-density lipoprotein cholesterol; WBC, white blood cell; MHR, monocyte/HDL-c; NHR, neutrophil/HDL-c; NLR, neutrophil/lymphocyte; PLR, platelet/lymphocyte. Bold values indicate statistically significance*.

### Evaluation of the Impact of MHR on MAFLD With T2DM

**Figure 4:**
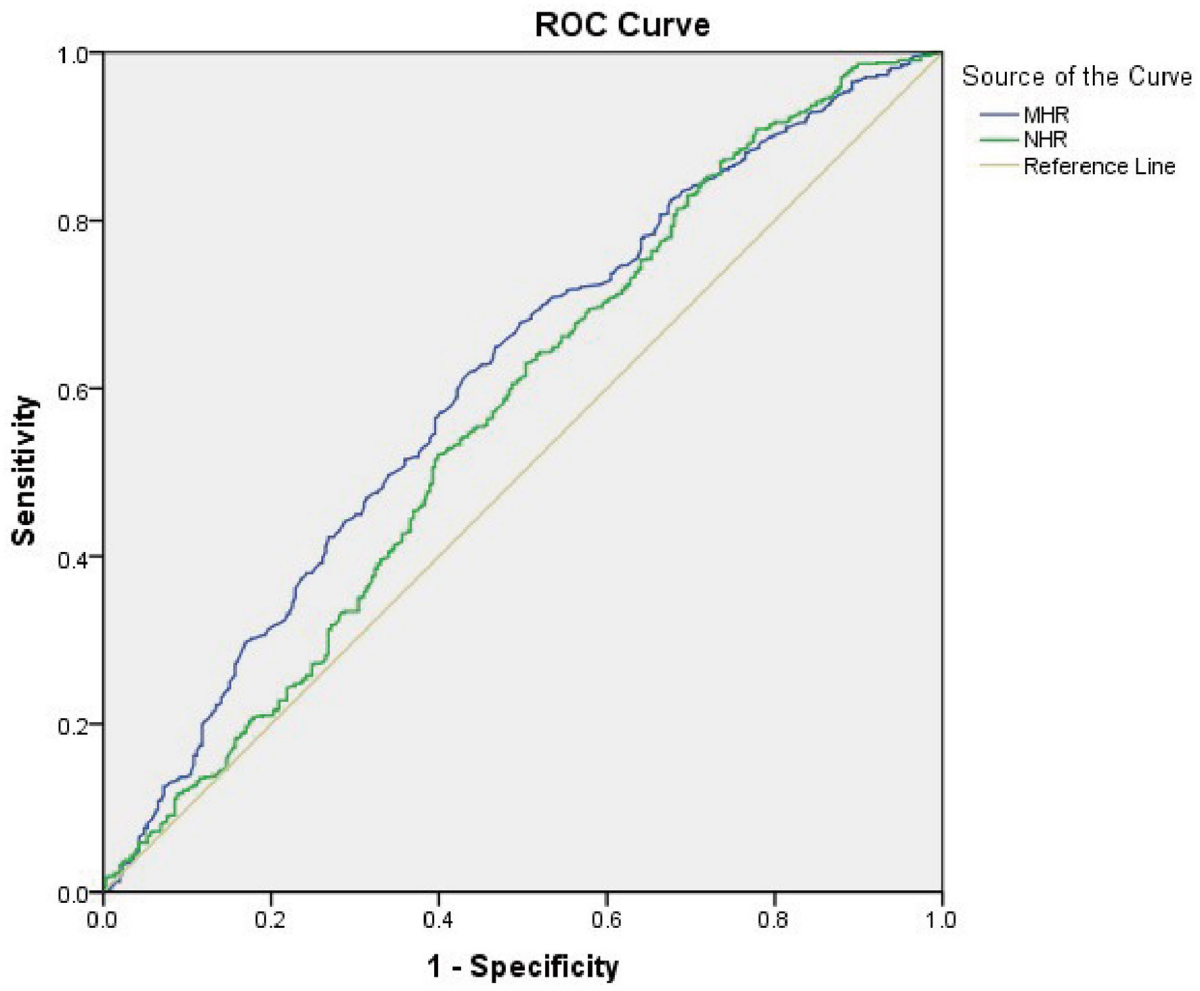
Receiver operating characteristic (ROC) curve analysis of monocyte to HDL cholesterol ratio (MHR) and neutrophil to HDL cholesterol ratio (NHR) to assess the accuracy of these parameters as a biomarker of MAFLD risk in T2DM patients. The area under the ROC curve (AUC) values in the MHR and NHR were 0.610 (95% confidence interval: 0.573–0.648) and 0.571 (95% confidence interval: 0.531–0.611), respectively.

As Figure 4 shows the performance for evaluating the endpoint among the inflammatory markers for MAFLD risk, the AUC of the marker is as follows: MHR 0.610 (95% CI: 0.573–0.648), NHR 0.571 (95% CI: 0.531–0.611), NLR 0.438 (95% CI: 0.400–0.477), and PLR 0.443 (95% CI: 0.404–0.482). The result demonstrated that the AUC assessed by MHR was larger than that of the other inflammatory markers (*P* < 0.01). The cutoff value of MHR was 0.388 with a sensitivity of 61.74% and a specificity of 56.54% (Table 4).

**Table 4:** ROC curve analysis of MHR in assessing MAFLD risk in patients with T2DM.

**Variables**	**AUC**	**95%CI**	**Sensitivity (%)**	**Specificity (%)**	**Cut-Off value**	**Youden Index**
MHR	0.610	0.573–0.648	61.74	56.54	0.388	0.183
NHR	0.571	0.531–0.611	86.98	26.47	2.029	0.135
NLR	0.438	0.400–0.477	99.73	0.33	0.574	0.001
PLR	0.443	0.404–0.482	0.40	100	318.036	0.004

*MHR, monocyte/HDL-c; NHR, neutrophil/HDL-c; NLR, neutrophil/lymphocyte; PLR, platelet/lymphocyte*.

Then, based on the cutoff point, the whole patients were separated into high MHR group and low MHR group, and the result showed that the high MHR group had a significantly higher level of height, weight, BMI, NC, WC, HC, DBP, VFA, SFA, fasting plasma insulin, fasting C-peptide, 2-h plasma insulin, 2-h C-peptide, HOMA-IR, ALT, γ-GGT, creatinine, uric acid, TG, WBC, neutrophil, lymphocyte, platelet, NHR, and NLR than the low MHR group, while HOMA-ISI, TCHOL, HDL-c, LDL-c, and PLR were reduced in the high MHR group than the low MHR group. The prevalence of MAFLD in the high MHR group was higher than that in the low MHR group (77.57 and 62.22%, respectively, Supplementary Table 2).

**Figure 5:**
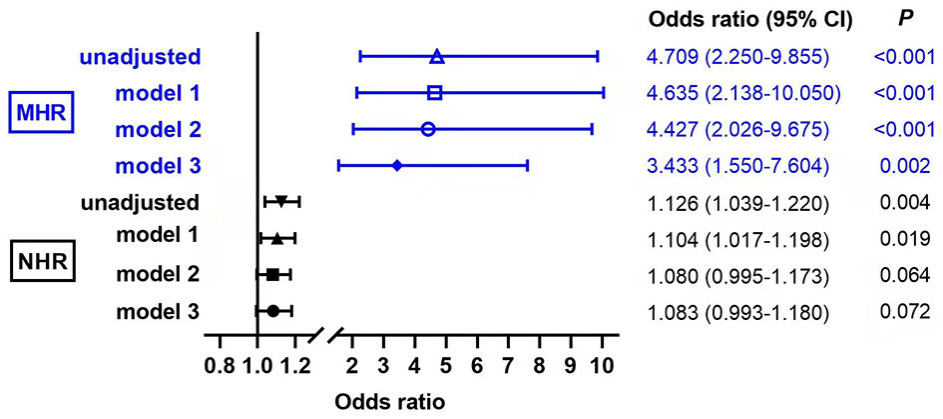
Evaluation of the impact of monocyte to HDL cholesterol ratio (MHR) and neutrophil to HDL cholesterol ratio (NHR) on MAFLD with T2DM by binary logistic regression analyses. Model 1: adjusted for age, gender; Model 2: adjusted for smoking, alcohol intake, hypertension history, in addition to model 1; Model 3: adjusted for use of antidiabetic drug, homeostasis model assessment of insulin resistance (HOMA-IR), total cholesterol (TC), homeostasis model assessment of insulin sensitivity index (HOMA-ISI), HbAlc in addition to model 2.

We further performed the binary logistic regression analyses, and the result showed that the risk of MAFLD significantly increased with the increasing of MHR (*P* < 0.01 in every model, Figure 5). In the base model, MHR was independently associated with MAFLD (*P* < 0.001). After adjusting for gender and age, MHR and MAFLD were independently correlated (*P* < 0.001 in model 1). After additional correction of smoking, alcohol intake, and hypertension history, MHR and MAFLD still showed an independent correlation (*P* < 0.001 in model 2). Furthermore, the MHR was also an independent determinant of MAFLD after further adjustment of usage of antidiabetic drug, HOMA-IR, TC, HOMA-ISI, and HbA1c (*P* = 0.002 in model 3). This indicates that the high MHR is independently associated with MAFLD in patients with T2DM. We also observed that NHR was independently associated with MAFLD in the unadjusted model (*P* = 0.004) and model 1 (*P* =0.019), while no significant correlation was found in models 2 and 3 (*P* = 0.064 and *P* = 0.072, respectively).

### The Clinical and Biochemical Characteristics According to MHR Quartiles

The study population was divided according to MHR quartiles: Q1 (MHR ≤ 0.31, *n* = 263), Q2 (0.31 < MHR ≤ 0.41, *n* = 266), Q3 (0.41 < MHR ≤ 0.56, *n* = 268), Q4 (MHR > 0.56, *n* = 254), as shown in Table 5. ANOVA revealed that groups with higher values of MHR had remarkably higher height, weight, BMI, NC, WC, HC, VFA, SFA, fasting plasma insulin, fasting C-peptide, 2-h plasma insulin, 2-h C-peptide, HOMA-IR, ALT, γ-GGT, creatinine, uric acid, TG, WBC, neutrophil, monocyte, lymphocyte, platelet, NHR, significantly lower age, HOMA-ISI, albumin, TCHOL, HDL-c, LDL-c, and PLR (*P* < 0.05).

**Table 5:** The clinical and biochemical characteristics according to MHR quartiles.

**Variables**	**Q1 (*n* = 263)**	**Q2 (*n* = 266)**	**Q3 *(n* = 268)**	**Q4 (*n* = 254)**	***P* value**
	**(MHR ≤ 0.31)**	**(0.31 < MHR ≤ 0.41)**	**(0.41 < MHR ≤ 0.56)**	**(MHR > 0.56)**	
Age (years)	**58.00 (52.00, 66.00)**	**58.00 (50.00, 66.00)**	**55.50 (46.00, 64.00)**	**54.00 (44.00, 62.25)**	**<0.001**
Male (*n*, %)	**110 (41.83)**	**151 (56.77)**	**171 (63.81)**	**211 (83.07)**	**<0.001**
Smoking (*n*, %)	**42 (15.97)**	**68 (25.56)**	**64 (23.88)**	**104 (40.94)**	**<0.001**
Alcohol intake (*n*, %)	25 (9.51)	36 (13.53)	46 (17.16)	32 (12.60)	0.075
Hypertension (*n*, %)	136 (51.71%)	151 (56.77)	142 (52.99)	142 (55.91)	0.612
History of CAD (*n*, %)	18 (6.84)	26 (9.77)	17 (6.34)	21 (8.27)	0.450
Dyslipidemia (*n*, %)	**161 (61.22)**	**210 (78.95)**	**233 (86.94)**	**238 (93.70)**	**<0.001**
Antidiabetic drug (*n*, %)	**229 (87.07)**	**213 (80.08)**	**215 (80.22)**	**195 (76.77)**	**0.023**
Height (cm)	**162.79 ± 8.48**	**165.88 ± 8.28**	**166.39 ± 8.23**	**169.40 ± 7.87**	**<0.001**
Weight (kg)	**62.90 (57.20, 70.00)**	**67.35 (61.15, 75.80)**	**69.55 (62.43, 77.08)**	**73.85 (65.65, 82.13)**	**<0.001**
BMI (kg/m^2^)	**24.00 (22.00, 26.20)**	**24.50 (22.70, 27.13)**	**25.20 (23.23, 27.20)**	**25.63 (23.40, 28.13)**	**<0.001**
NC (cm)	**36.00 (34.00, 39.00)**	**38.00 (36.00, 41.00)**	**39.00 (36.00, 41.38)**	**40.00 (37.88, 42.00)**	**<0.001**
WC (cm)	**87.00 (82.00, 93.00)**	**91.00 (85.00, 97.00)**	**93.00 (86.50, 98.00)**	**94.00 (88.00, 100.00)**	**<0.001**
HC (cm)	**96.00 (92.00, 100.00)**	**97.00 (93.00, 101.00)**	**97.00 (93.00, 103.00)**	**98.00 (94.00, 103.00)**	**<0.001**
SBP (mmHg)	127.00 (117.00, 139.00)	128.00 (114.00, 140.00)	126.00 (116.00, 138.00)	129.00 (117.00, 140.00)	0.760
DBP (mmHg)	74.50 ± 10.54	74.00 ± 10.92	74.52 ± 9.92	76.21 ± 9.78	0.080
MAP (mmHg)	92.59 ± 11.03	92.05 ± 11.97	92.42 ± 10.90	93.92 ± 10.74	0.251
VFA (cm^2^)	**78.02 ± 37.32**	**91.33 ± 32.63**	**95.07 ± 36.11**	**105.09 ± 41.14**	**<0.001**
SFA (cm^2^)	**174.11 ± 68.95**	**180.16 ± 57.07**	**184.32 ± 63.35**	**197.61 ± 69.94**	**<0.001**
Fasting plasma glucose (mmol/L)	9.68 (7.09, 12.46)	9.69 (7.99, 12.52)	10.06 (7.77, 12.91)	10.03 (8.04, 12.93)	0.257
Fasting plasma insulin (μIU/mL)	**6.89 (3.60, 10.33)**	**6.79 (4.12, 10.66)**	**7.63 (4.59, 11.21)**	**9.31 (5.29, 12.11)**	**<0.001**
Fasting C-peptide (ng/mL)	**2.07 ± 1.11**	**2.40 ± 0.95**	**2.52 ± 1.06**	**2.74 ± 1.13**	**<0.001**
2h plasma glucose (mmol/L)	18.95 ± 5.56	18.87 ± 5.17	19.46 ± 5.10	19.48 ± 5.01	0.383
2h plasma insulin (μIU/mL)	**31.96 (16.61, 44.83)**	**27.08 (15.25, 39.59)**	**30.78 (16.14, 46.59)**	**34.17 (19.66, 46.98)**	**0.037**
2h C-peptide (ng/mL)	**4.31 (2.87, 6.62)**	**4.18 (3.29, 6.15)**	**4.59 (3.22, 6.85)**	**5.09 (3.51, 7.17)**	**0.010**
HbA1c (%)	9.30 (7.50, 11.00)	9.50 (8.00, 10.90)	9.65 (8.10, 11.10)	9.48 (8.00, 11.03)	0.425
HOMA-IR	**2.76 (1.64, 4.28)**	**3.07 (1.77, 4.66)**	**3.43 (1.94, 5.17)**	**3.89 (2.37, 5.43)**	**<0.001**
HOMA-ISI	**0.41 (0.25, 0.61)**	**0.38 (0.23, 0.58)**	**0.35 (0.19, 0.54)**	**0.30 (0.18, 0.44)**	**<0.001**
ALT (U/L)	**19.50 (13.20, 29.70)**	**19.85 (13.30, 32.23)**	**22.10 (15.00, 37.88)**	**24.00 (16.98, 43.85)**	**<0.001**
AST (U/L)	17.30 (14.00, 24.80)	17.00 (13.00, 22.87)	17.85 (13.35, 24.23)	18.50 (13.58, 25.53)	0.309
ALP (U/L)	71.00 (59.00, 86.00)	70.50 (57.00, 90.00)	70.00 (58.00, 83.00)	71.50 (57.00, 86.00)	0.905
γ-GGT (U/L)	**26.00 (16.00, 42.00)**	**26.00 (19.00, 41.00)**	**29.00 (21.00, 47.00)**	**35.00 (25.00, 60.25)**	**<0.001**
Albumin (g/L)	**41.56 ± 5.91**	**40.25 ± 3.86**	**40.27 ± 3.83**	**40.02 ± 3.70**	**0.025**
Blood urea nitrogen (mmol/L)	5.30 ± 1.48	5.69 ± 2.00	5.50 ± 1.70	5.75 ± 2.31	0.239
Creatinine (μmol/L)	**57.00 (47.80, 66.10)**	**58.70 (48.58, 68.58)**	**60.15 (50.13, 70.40)**	**62.80 (53.80, 71.83)**	**0.001**
Uric acid (μmol/L)	**270.02 ± 79.93**	**288.33 ± 90.26**	**295.64 ± 82.77**	**319.71 ± 88.32**	**<0.001**
TCHOL (mmol/L)	**5.04 (4.31, 5.78)**	**4.89 (4.26, 5.64)**	**4.77 (4.15, 5.44)**	**4.49 (3.88, 5.41)**	**<0.001**
TG (mmol/L)	**1.53 (1.06, 2.27)**	**1.91 (1.34, 2.72)**	**2.00 (1.49, 2.98)**	**2.41 (1.67, 3.60)**	**<0.001**
HDL-c (mmol/L)	**1.34 (1.13, 1.60)**	**1.12 (1.01, 1.27)**	**0.99 (0.86, 1.15)**	**0.84 (0.70, 0.98)**	**<0.001**
LDL-c (mmol/L)	**2.94 ± 0.99**	**2.86 ± 0.90**	**2.85 ± 0.85**	**2.61 ± 0.93**	**<0.001**
WBC (*10^9^/L)	**5.00 (4.20, 6.10)**	**5.70 (5.00, 6.60)**	**6.00 (5.20, 7.10)**	**7.10 (6.10, 8.43)**	**<0.001**
Neutrophil (*10^9^/L)	**2.80 (2.20, 3.70)**	**3.20 (2.68, 3.93)**	**3.30 (2.70, 4.10)**	**4.00 (3.28, 4.93)**	**<0.001**
Monocyte (*10^9^/L)	**0.30 (0.30, 0.40)**	**0.40 (0.40, 0.50)**	**0.50 (0.40, 0.50)**	**0.60 (0.50, 0.70)**	**<0.001**
Lymphocyte (*10^9^/L)	**1.73 ± 0.55**	**1.94 ± 0.58**	**2.03 ± 0.65**	**2.29 ± 0.91**	**<0.001**
Platelet (*10^9^/L)	**187.20 ± 52.61**	**184.18 ± 53.81**	**197.68 ± 56.47**	**207.34 ± 56.02**	**<0.001**
NHR	**2.12 (1.58, 2.90)**	**2.86 (2.31, 3.42)**	**3.30 (2.71, 4.16)**	**4.67 (3.86, 6.00)**	**<0.001**
NLR	1.65 (1.25, 2.20)	1.72 (1.33, 2.36)	1.65 (1.29, 2.25)	1.78 (1.40, 2.45)	0.066
PLR	**110.50 (85.00, 138.33)**	**94.89 (72.36, 125.14)**	**99.37 (77.07, 124.33)**	**92.81 (72.03, 119.02)**	**<0.001**

*CAD, coronary artery disease; BMI, body mass index; NC, neck circumference; WC, waist circumference; HC, hip circumference; SBP, systolic blood pressure; DBP, diastolic blood pressure; VFA, visceral fat area; SFA, subcutaneous fat area; HOMA-IR, homeostasis model assessment of insulin resistance; HOMA-ISI, homeostasis model assessment of insulin sensitivity index; ALT, alanine aminotransferase; AST, aspartate aminotransferase; ALP, alkaline phosphatase; γ-GGT, gamma-glutamyl transferase; TCHOL, total cholesterol; TG, triglyceride; HDL-c, high-density lipoprotein cholesterol; LDL-c, low-density lipoprotein cholesterol; WBC, white blood cell; MHR, monocyte/HDL-c; NHR, neutrophil/HDL-c; NLR, neutrophil/lymphocyte; PLR, platelet/lymphocyte. Bold values indicate statistically significance*.

### The Prevalence of MAFLD Among Different Quartiles of MHR

**Figure 6:**
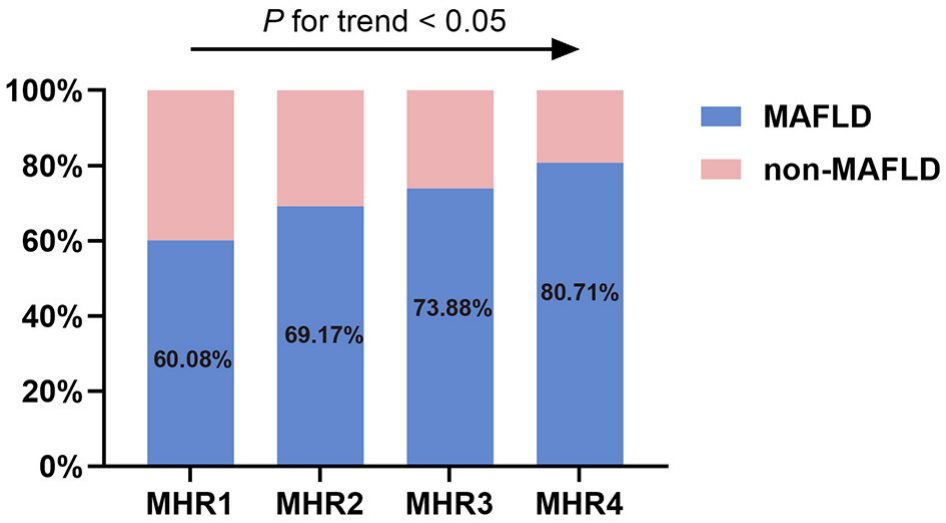
The prevalence of MAFLD in T2DM patients among different quartiles of monocyte to HDL cholesterol ratio (MHR).

**Figure 7:**
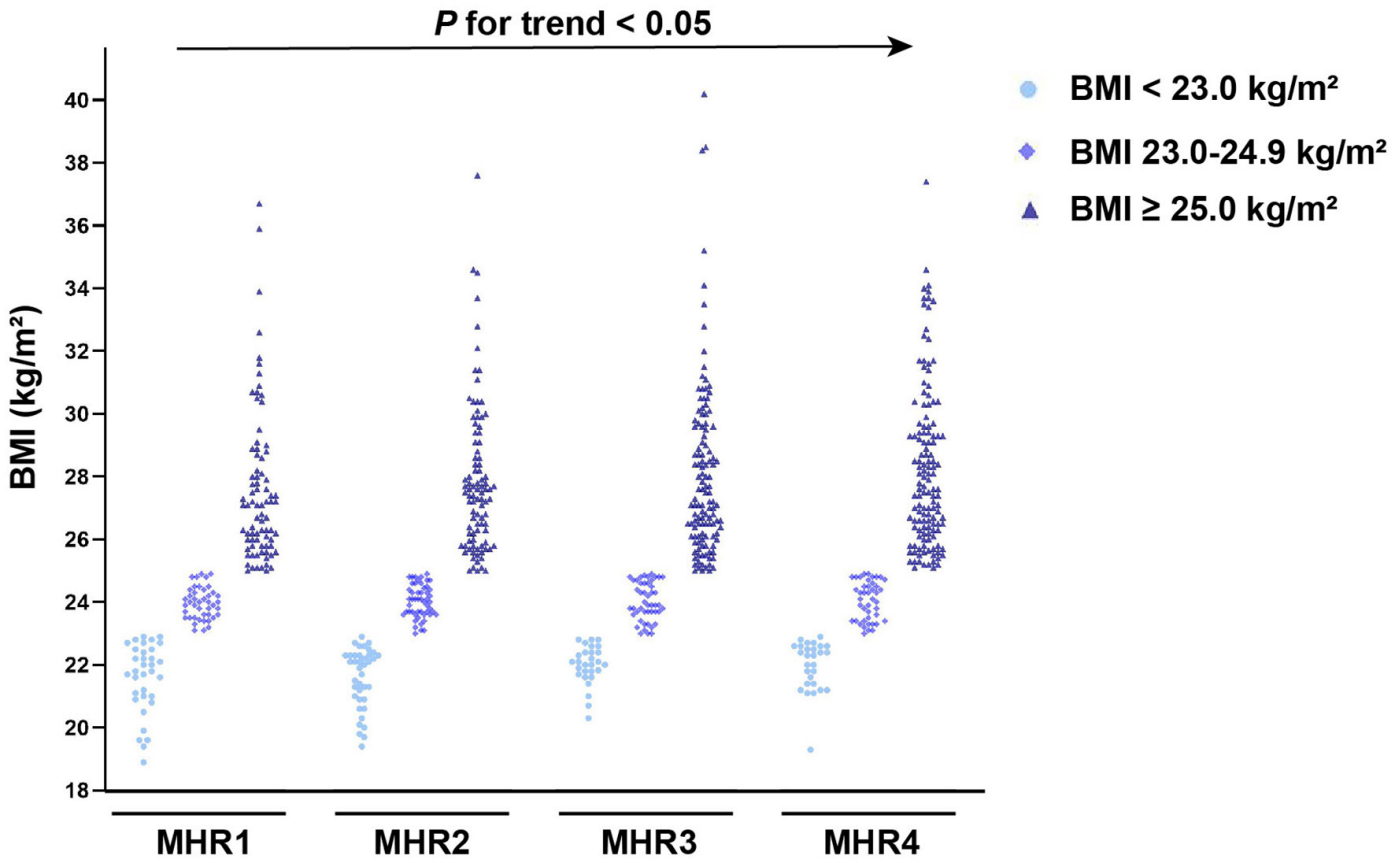
The number of MAFLD patients with T2DM based on body mass index (BMI) among different quartiles of monocyte to HDL cholesterol ratio (MHR).

As illustrated in Figure 6, groups with higher values of MHR had a significantly higher prevalence of MAFLD (*P* < 0.05). In each quartile, as shown in Figure 7, when the patients with MAFLD were divided into lean MAFLD group, overweight MAFLD group, and obese MAFLD group according to BMI, the number of obese MAFLD patients increased as the MHR level increased.

### Distribution of Metabolic Dysfunction in Patients With MAFLD Among Different Quartiles of MHR

**Figure 8:**
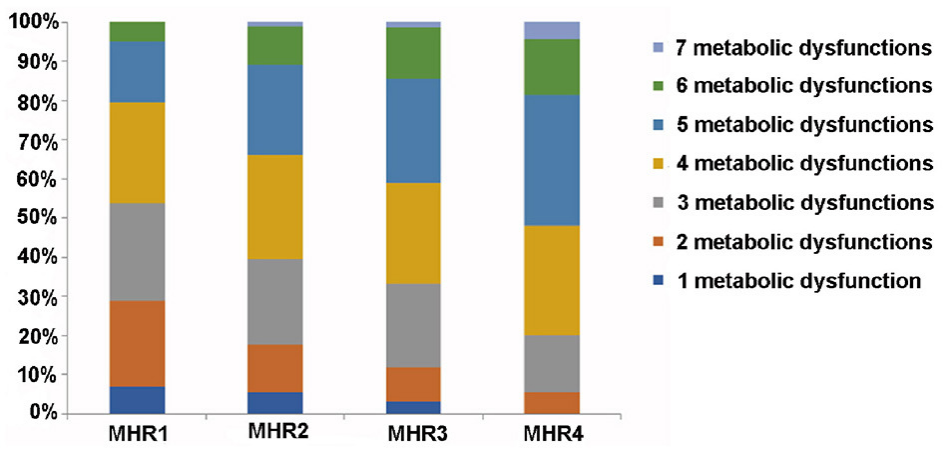
Distribution of metabolic dysfunction in MAFLD patients among different quartiles of monocyte to HDL cholesterol ratio (MHR). Metabolic dysfunction indicators: waist circumference (WC) ≥ 90 cm for men and ≥ 80 cm for women; systolic blood pressure (SBP) ≥ 130 mmHg or diastolic blood pressure (DBP) ≥85 mmHg or treatment of previously diagnosed hypertension; TG levels ≥ 1.70 mmol/L or specific treatment for this lipid abnormalities; high-density lipoprotein cholesterol levels (HDL-c) of < 1.0 mmol/L in men and < 1.3 mmol/L in women or specific treatment for this lipid abnormalities; fasting plasma glucose (FPG) of ≥ 5.60 mmol/L or previously diagnosed T2DM; uric acid (UA) levels of ≥420 μmol/L or specific treatment for this abnormalities; urinary microalbumin (uMA) > 30 mg/L or MA/UCREA > 30 mg/L.

We further analyzed the distribution of metabolic dysfunction in patients with MAFLD among different quartiles of MHR, and the result displayed that with the increase of MHR, the percentage of patients with MAFLD who had more than four metabolic dysfunction indicators increased, which was 46.39, 60.52, 66.79, and 79.91%, respectively, in each quartile (Figure 8).

## Discussion

The prevalence of NAFLD in the T2DM population has been proved to reach approximately 40–70% which is higher than that in the general population (Younossi et al., 2019; Mantovani et al., 2020). The finding in this study is consistent with the previous reports, and the prevalence of MAFLD is reaching 70.88%. The pathogenesis of the two comorbid disorders of NAFLD and T2DM has been studied extensively, while the exact molecular mechanisms are still undiscovered (Wu et al., 2017). It is well-recognized that IR is the core to the pathogenesis of NAFLD and T2DM (Wu et al., 2017). IR could lead to hyperglycemia and reactive hyperinsulinemia, which in turn could cause lipid accumulation, and finally affects lipid metabolism in the liver (Li P. et al., 2020). Simultaneously, the current result shows that the MAFLD group has higher levels of BMI, NC, WC, HC, VFA, and SFA than the non-MAFLD group, which indicates that patients with T2DM in overweight and obese groups are more likely to be combined with MAFLD. Previous report clarified that obesity could increase the risk of developing IR, T2DM, dyslipidemia, hypertension, and NAFLD (Jung and Choi, 2014). In addition, many studies suggest that chronic inflammation in adipose tissue might play a significant role in the development of obesity-related metabolic dysfunction (Zatterale et al., 2019).

This study observes the effect of MHR on the assessment of MAFLD in T2DM. Our findings manifested that the MHR was higher in T2DM patients with MAFLD, compared with the control group. Concurrently, when the study population was divided according to MHR quartiles, the prevalence of MAFLD increased as the values of MHR increased. Meanwhile, in each quartile, the percentage of patients with obese MAFLD increased as the MHR level increased. In patients with MAFLD, Pearson or Spearman correlation analysis demonstrated that the MHR is positively related to BMI, NC, WC, HC, VFA, and HOMA-IR. In recent years, MHR was confirmed to be a novel maker with the integration of pro-inflammatory and anti-inflammatory indices, and it owned comparatively higher clinic practical values since it is convenient to obtain (Chen et al., 2019). Wang et al. demonstrated a linear relation between MHR levels and the odds of ischemic stroke in a large community-based population, and they also found that MHR could be a clinical indicator in risk stratification in subjects with ischemic stroke (Wang et al., 2019). In a metabolic syndrome-related study, the scholars investigated and found out that patients with metabolic syndrome had higher MHR values than healthy controls; moreover, MHR could be an inflammatory marker to evaluate disease severity (Uslu et al., 2018). Cetin et al. demonstrated that MHR might be an independent predictor of the severity of coronary artery disease and future cardiovascular events in patients with acute coronary syndrome (Cetin et al., 2016).

The activation of monocytes and their differentiated forms into lipid-laden macrophages play an important role in promoting immune defenses in patients with chronic inflammatory (Usta et al., 2018). Meanwhile, the activation of monocytes and their differentiated forms into lipid-laden macrophages could be regulated by inflammatory cytokines (Akboga et al., 2016). Previous study shows that the monocyte count is an independent predictor of plaque formation and progression in atherosclerosis (Johnsen et al., 2005). Study also demonstrated that the M1 type macrophage/M2 type macrophage ratio was increased during the progression of liver disease (Ziolkowska et al., 2021). On the contrary, HDL-c has anti-inflammatory, antioxidant, and antithrombotic effects, which has the ability to counteract macrophages migration and remove cholesterol from these cells (Akboga et al., 2016; Usta et al., 2018). HDL-c molecules also play a suppressive role in the control of monocyte activation, as well as in the proliferation and differentiation of the progenitor cells of monocytes, as reported in previous study (Usta et al., 2018). Therefore, monocytes play a pro-inflammatory role, while HDL-c shows a reversal factor during this process (Yilmaz and Kayançiçek, 2018). Higher monocyte counts and lower LDL-c levels act as indirect indicators of inflammation and development of atherosclerosis (Usta et al., 2018). Actually, the relationship between monocyte counts and HDL-c provides a better understanding of inflammation. MAFLD is recognized as the liver disease component of metabolic syndrome, which is mainly associated with obesity, IR, T2DM, and inflammation (Li H. et al., 2020). In this study, the higher MHR in MAFLD patients with T2DM has been confirmed. The ROC curve showed that the evaluative value of MHR for MAFLD risk was 0.610. For further study, with binary logistic regression analyses of MAFLD as a dependent variable, the relationship between MHR and MAFLD was significant. After adjusting for many factors, the relationship still existed. It also demonstrated that with the increasing of the MHR, the percentage of patients with MAFLD who had more than four metabolic dysfunction indicators increased. Above all, the MHR has the advantage as an evaluating indicator of MAFLD in T2DM patients, which is reported for the first time as far as we know.

We also found that the NHR was higher in T2DM patients with MAFLD than that in the non-MAFLD group. Kou et al. recently demonstrated that the NHR was closely related to CAD, and it was an independent predictor of severe coronary stenosis (Huang et al., 2020; Kou et al., 2021). Previous study showed that the NHR had a strong predictive value for predicting metabolic syndrome (Chen et al., 2020). In this study, the ROC curve showed that the evaluative value of the NHR for MAFLD risk was 0.571, with a sensitivity of 86.98% and a specificity of 26.47% only, which was inferior to that of MHR. The increased number of lymphocytes was found in the MAFLD group. This is similar to the previous report that the percentage of lymphocytes is independently and positively correlated with MAFLD (Li H. et al., 2020). ROC curve of the value of lymphocyte for predicting MAFLD risk was analyzed (data were not shown), and the result was also inferior to that of MHR.

### Limitation

Several limitations exist in this study. First, this study was a retrospective analysis based on prospectively collected data from a single center in the Chinese population. Second, the golden criteria of MAFLD diagnosis were based on histological examination and imaging techniques. However, these two techniques are invasive, expensive, and unfeasible in clinical work. Third, in this study, the relationship between the MHR level and the severity of MAFLD was not clarified. Further prospective studies should be performed to investigate whether MHR would be an evaluating indicator of improving MAFLD.

## Conclusion

The MHR is a convenient, simple and cost-effective, parameter that could be used for assessing MAFLD in T2DM. T2DM patients with a higher MHR have more possibility to be diagnosed as MAFLD. Therefore, more attention should be given to the indicator in the examination of T2DM.

## Data Availability Statement

The original contributions presented in the study are included in the article/Supplementary Material, further inquiries can be directed to the corresponding author/s.

## Ethics Statement

The study protocol was approved by the Human Research Ethics Committee of the Affiliated Hospital of Jiangsu University. The patients/participants provided their written informed consent to participate in this study.

## Author Contributions

JJ and GY participated in the study design. JJ, RL, WW, XY, YS, ZZ, and CC were involved in the conduct of the study and data collection. JJ, RL, WW, FY, and ZC made contributions to data analysis and result interpretation. JJ, RL, CW, DW, and LY wrote and modified the manuscript and prepared tables and figures. All authors read and approved the final manuscript.

## Funding

This study was supported by the National Natural Science Foundation of China (81870548, 81570721, and 81500351), the Social Development Project of Jiangsu Province (BE2018692), the Natural Science Foundation of Jiangsu Province (BK20191222), the Youth Medical Talent Project of Jiangsu Province (QNRC2016842), the Jiangsu University Affiliated Hospital 5123 Talent Plan (51232017305), the sixth 169 Talent Project of Zhenjiang, the Science and Technology Commission of Zhenjiang City (FZ2020038), Doctoral Research Initiation Fund (jdfyRC2020010), and Clinical Medical Science and Technology Development Foundation of Jiangsu University (JLY2021209).

## Conflict of Interest

The authors declare that the research was conducted in the absence of any commercial or financial relationships that could be construed as a potential conflict of interest.

## Publisher's Note

All claims expressed in this article are solely those of the authors and do not necessarily represent those of their affiliated organizations, or those of the publisher, the editors and the reviewers. Any product that may be evaluated in this article, or claim that may be made by its manufacturer, is not guaranteed or endorsed by the publisher.

## Abbreviations

T2DM: type 2 diabetes mellitus
CAD: coronary artery disease
BMI: body mass index
NC: neck circumference
WC: waist circumference
HC: hip circumference
SBP: systolic blood pressure
DBP: diastolic blood pressure
MAP: mean arterial pressure
VFA: visceral fat area
SFA: subcutaneous fat area
HOMA-IR: homeostasis model assessment of insulin resistance
HOMA-ISI: homeostasis model assessment of insulin sensitivity index
ALT: alanine aminotransferase
AST: aspartate aminotransferase
ALP: alkaline phosphatase
γ-GGT: gamma-glutamyl transferase
TCHOL: total cholesterol
TG: triglyceride
HDL-c: high-density lipoprotein cholesterol
LDL-c: low-density lipoprotein cholesterol
WBC: white blood cell
MHR: monocyte/HDL-c
NHR: neutrophil/HDL-c
NLR: neutrophil/lymphocyte
PLR: platelet/lymphocyte
NFS: non-alcoholic fatty liver disease fibrosis score.

## References

[B1] Akboga M. K. Balci K. G. Maden O. Ertem A. G. Kirbas O. Yayla C.. (2016). Usefulness of monocyte to HDL-cholesterol ratio to predict high SYNTAX score in patients with stable coronary artery disease. Biomark. Med. 10, 375–383. 10.2217/bmm-2015-0050 26999570

[B2] American Diabetes Association (2021). 2. classification and diagnosis of diabetes: standards of medical care in diabetes-2021. Diabetes Care. 44, S15–S33. 10.2337/dc21-S002 33298413

[B3] Blanquet M. Legrand A. Pélissier A. Mourgues C. (2019). Socio-economics status and metabolic syndrome: a meta-analysis. Diabetes Metab. Syndr. 13, 1805–1812. 10.1016/j.dsx.2019.04.003 31235098

[B4] Bril F. McPhaul M. J. Caulfield M. P. Clark V. C. Soldevilla-Pico C. Firpi-Morell R. J.. (2020). Performance of plasma biomarkers and diagnostic panels for nonalcoholic steatohepatitis and advanced fibrosis in patients with type 2 diabetes. Diabetes Care. 43, 290–297. 10.2337/dc19-1071 31604692

[B5] Cetin M. S. Ozcan Cetin E. H. Kalender E. Aydin S. Topaloglu S. Kisacik H. L.. (2016). Monocyte to HDL cholesterol ratio predicts coronary artery disease severity and future major cardiovascular adverse events in acute coronary syndrome. Heart Lung Circ. 25, 1077–1086. 10.1016/j.hlc.2016.02.023 27118231

[B6] Chen J. W. Li C. Liu Z. H. Shen Y. Ding F. H. Shu X. Y.. (2019). The role of monocyte to high-density lipoprotein cholesterol ratio in prediction of carotid intima-media thickness in patients with type 2 diabetes. Front Endocrinol (Lausanne). 10:191. 10.3389/fendo.2019.00191 31019490 PMC6458254

[B7] Chen T. Chen H. Xiao H. Tang H. Xiang Z. Wang X.. (2020). Comparison of the value of neutrophil to high-density lipoprotein cholesterol ratio and lymphocyte to high-density lipoprotein cholesterol ratio for predicting metabolic syndrome among a population in the Southern Coast of China. Diabetes Metab. Syndr. Obes. 13, 597–605. 10.2147/DMSO.S238990 32184639 PMC7053653

[B8] Eslam M. Sanyal A. J. George J. (2020). MAFLD: a consensus-driven proposed nomenclature for metabolic associated fatty liver disease. Gastroenterology. 158, 1999–2014.e1991. 10.1053/j.gastro.2019.11.312 32044314

[B9] Ferguson D. Finck B. N. (2021). Emerging therapeutic approaches for the treatment of NAFLD and type 2 diabetes mellitus. Nat. Rev. Endocrinol. 17, 484–495. 10.1038/s41574-021-00507-z 34131333 PMC8570106

[B10] Han Y. H. Lee K. Saha A. Han J. Choi H. Noh M.. (2021). Specialized proresolving mediators for therapeutic interventions targeting metabolic and inflammatory disorders. Biomol. Ther. (Seoul) 29, 455–464. 10.4062/biomolther.2021.094 34162770 PMC8411019

[B11] Huang J. B. Chen Y. S. Ji H. Y. Xie W. M. Jiang J. Ran L. S.. (2020). Neutrophil to high-density lipoprotein ratio has a superior prognostic value in elderly patients with acute myocardial infarction: a comparison study. Lipids Health Dis. 19:59. 10.1186/s12944-020-01238-2 32247314 PMC7126405

[B12] Johnsen S. H. Fosse E. Joakimsen O. Mathiesen E. B. Stensland-Bugge E. Njølstad I.. (2005). Monocyte count is a predictor of novel plaque formation: a 7-year follow-up study of 2610 persons without carotid plaque at baseline the Tromsø Study. Stroke. 36, 715–719. 10.1161/01.STR.0000158909.07634.83 15746459

[B13] Jung U. J. Choi M. S. (2014). Obesity and its metabolic complications: the role of adipokines and the relationship between obesity, inflammation, insulin resistance, dyslipidemia and nonalcoholic fatty liver disease. Int. J. Mol. Sci. 15, 6184–6223. 10.3390/ijms15046184 24733068 PMC4013623

[B14] Kou T. Luo H. Yin L. (2021). Relationship between neutrophils to HDL-C ratio and severity of coronary stenosis. BMC Cardiovasc. Disord. 21:127. 10.1186/s12872-020-01771-z 33676400 PMC7936429

[B15] Kumar S. Duan Q. Wu R. Harris E. N. Su Q. (2021). Pathophysiological communication between hepatocytes and non-parenchymal cells in liver injury from NAFLD to liver fibrosis. Adv. Drug Deliv. Rev. 176:113869. 10.1016/j.addr.2021.113869 34280515 PMC11792083

[B16] Li H. Guo M. An Z. Meng J. Jiang J. Song J.. (2020). Prevalence and risk factors of metabolic associated fatty liver disease in Xinxiang, China. Int. J. Environ. Res. Public Health 17:1818. 10.3390/ijerph17061818 32168920 PMC7143027

[B17] Li P. Fan C. Cai Y. Fang S. Zeng Y. Zhang Y.. (2020). Transplantation of brown adipose tissue up-regulates miR-99a to ameliorate liver metabolic disorders in diabetic mice by targeting NOX4. Adipocyte 9, 57–67. 10.1080/21623945.2020.1721970 32000567 PMC6999837

[B18] Lima W. G. Martins-Santos M. E. Chaves V. E. (2015). Uric acid as a modulator of glucose and lipid metabolism. Biochimie 116, 17–23. 10.1016/j.biochi.2015.06.025 26133655

[B19] Loomba R. Friedman S. L. Shulman G. I. (2021). Mechanisms and disease consequences of nonalcoholic fatty liver disease. Cell. 184, 2537–2564. 10.1016/j.cell.2021.04.015 33989548 PMC12168897

[B20] Mantovani A. Scorletti E. Mosca A. Alisi A. Byrne C. D. Targher G. (2020). Complications, morbidity and mortality of nonalcoholic fatty liver disease. Metabolism 111s:154170. 10.1016/j.metabol.2020.154170 32006558

[B21] Nasr P. Fredrikson M. Ekstedt M. Kechagias S. (2020). The amount of liver fat predicts mortality and development of type 2 diabetes in non-alcoholic fatty liver disease. Liver Int. 40, 1069–1078. 10.1111/liv.14414 32087038

[B22] Osonoi T. Gouda M. Kubo M. Arakawa K. Hashimoto T. Abe M. (2018). Effect of canagliflozin on urinary albumin excretion in japanese patients with type 2 diabetes mellitus and microalbuminuria: a pilot study. Diabetes Technol. Ther. 20, 681–688. 10.1089/dia.2018.0169 30096243 PMC6161332

[B23] Powell E. E. Wong V. W. Rinella M. (2021). Non-alcoholic fatty liver disease. Lancet 397, 2212–2224. 10.1016/S0140-6736(20)32511-3 33894145

[B24] Sakurai Y. Kubota N. Yamauchi T. Kadowaki T. (2021). Role of Insulin Resistance in MAFLD. Int. J. Mol. Sci. 22:4156. 10.3390/ijms22084156 33923817 PMC8072900

[B25] Shin D. Lee K. W. (2021). High pre-pregnancy BMI with a history of gestational diabetes mellitus is associated with an increased risk of type 2 diabetes in Korean women. PLoS ONE 16:e0252442. 10.1371/journal.pone.0252442 34086709 PMC8177465

[B26] Tacke F. Weiskirchen R. (2021). Non-alcoholic fatty liver disease (NAFLD)/non-alcoholic steatohepatitis (NASH)-related liver fibrosis: mechanisms, treatment and prevention. Ann. Transl. Med. 9:729. 10.21037/atm-20-4354 33987427 PMC8106094

[B27] Targher G. Corey K. E. Byrne C. D. Roden M. (2021). The complex link between NAFLD and type 2 diabetes mellitus - mechanisms and treatments. Nat. Rev. Gastroenterol. Hepatol. 18, 599-612. 10.1038/s41575-021-00448-y 33972770

[B28] Uslu A. U. Sekin Y. Tarhan G. Canakci N Gunduz M. Karagulle M. (2018). Evaluation of monocyte to high-density lipoprotein cholesterol ratio in the presence and severity of metabolic syndrome. Clin. Appl. Thromb. Hemost. 24, 828–833. 10.1177/1076029617741362 29212375 PMC6714883

[B29] Usta A. Avci E. Bulbul C. B. Kadi H. Adali E. (2018). The monocyte counts to HDL cholesterol ratio in obese and lean patients with polycystic ovary syndrome. Reprod. Biol. Endocrinol. 16:34. 10.1186/s12958-018-0351-0 29631598 PMC5891948

[B30] Wang H. Y. Shi W. R. Yi X. Zhou Y. P. Wang Z. Q. Sun Y. X. (2019). Assessing the performance of monocyte to high-density lipoprotein ratio for predicting ischemic stroke: insights from a population-based Chinese cohort. Lipids Health Dis. 18:127. 10.1186/s12944-019-1076-6 31142338 PMC6542056

[B31] Wang X. Rao H. Zhao J. Wee A. Li X. Fei R.. (2020). STING expression in monocyte-derived macrophages is associated with the progression of liver inflammation and fibrosis in patients with nonalcoholic fatty liver disease. Lab. Invest. 100, 542–552. 10.1038/s41374-019-0342-6 31745210

[B32] Wu H. Zhang T. Pan F. Steer C. J. Li Z. Chen X.. (2017). MicroRNA-206 prevents hepatosteatosis and hyperglycemia by facilitating insulin signaling and impairing lipogenesis. J. Hepatol. 66, 816–824. 10.1016/j.jhep.2016.12.016 28025059 PMC5568011

[B33] Yilmaz M. Kayançiçek H. (2018). A new inflammatory marker: elevated monocyte to HDL cholesterol ratio associated with smoking. J. Clin. Med. 7:76. 10.3390/jcm7040076 29642607 PMC5920450

[B34] Yki-Järvinen H. Luukkonen P. K. Hodson L. Moore J. B. (2021). Dietary carbohydrates and fats in nonalcoholic fatty liver disease. Nat. Rev. Gastroenterol. Hepatol. 18, 770-786. 10.1038/s41575-021-00472-y 34257427

[B35] Younossi Z. M. Golabi P. de Avila L. Paik J. M. Srishord M. Fukui N.. (2019). The global epidemiology of NAFLD and NASH in patients with type 2 diabetes: a systematic review and meta-analysis. J. Hepatol. 71, 793–801. 10.1016/j.jhep.2019.06.021 31279902

[B36] Zatterale F. Longo M. Naderi J. Raciti G. A. Desiderio A. Miele C.. (2019). Chronic adipose tissue inflammation linking obesity to insulin resistance and type 2 diabetes. Front. Physiol. 10:1607. 10.3389/fphys.2019.01607 32063863 PMC7000657

[B37] Zhang D. P. Baituola G. Wu T. T. Chen Y. Hou X. G. Yang Y.. (2020). An elevated monocyte-to-high-density lipoprotein-cholesterol ratio is associated with mortality in patients with coronary artery disease who have undergone PCI. Biosci. Rep. 40:BSR20201108. 10.1042/BSR20201108 32766711 PMC7432996

[B38] Zhang Y. Li K. Kong A. Zhou Y. Chen D. Gu J.. (2021). Dysregulation of autophagy acts as a pathogenic mechanism of non-alcoholic fatty liver disease (NAFLD) induced by common environmental pollutants. Ecotoxicol. Environ. Saf. 217:112256. 10.1016/j.ecoenv.2021.112256 33901779

[B39] Zhou J. Bai L. Zhang X. J. Li H. Cai J. (2021). Nonalcoholic fatty liver disease and cardiac remodeling risk: pathophysiological mechanisms and clinical implications. Hepatology 74, 2839–2847. 10.1002/hep.32072 34309877

[B40] Ziolkowska S. Binienda A. Jablkowski M. Szemraj J. Czarny P. (2021). The interplay between insulin resistance, inflammation, oxidative stress, base excision repair and metabolic syndrome in nonalcoholic fatty liver disease. Int. J. Mol. Sci. 22:11128. 10.3390/ijms222011128 34681787 PMC8537238

