# Supplementary material for: Monocyte to High-Density Lipoprotein Cholesterol Ratio at the Nexus of Type 2 Diabetes Mellitus Patients With Metabolic-Associated Fatty Liver Disease
Source: Front Physiol. 2021 Dec 17;12:762242. doi: 10.3389/fphys.2021.762242 (PMC8718696; doi:10.3389/fphys.2021.762242)
Supplement: Supplementary file 1 [file Table_1.docx]

Supplementary Table 1. The clinical, body composition and metabolic parameters of all subjects.

| Variables | Total patients (n=1051) |
| --- | --- |
| **Demographic Parameters** |  |
| Age (years) | 57.00(48.00,64.00) |
| Male (n,%) | 643(61.18) |
| Smoking (n,%) | 278(26.45) |
| Alcohol intake (n,%) | 139(13.23) |
| Hypertension (n,%) | 571(54.33) |
| History of CAD (n,%) | 82(7.80) |
| Dyslipidemia (n,%) | 842(80.11) |
| Antidiabetic drug (n,%) | 852(81.07) |
| **Anthropometric Parameters** |  |
| Height (cm) | 166.00(160.00,172.00) |
| Weight (kg) | 68.00(61.10,76.90) |
| BMI (kg/m^2^) | 24.80(22.80,27.20) |
| NC (cm) | 38.61±4.76 |
| WC (cm) | 91.39±9.65 |
| HC (cm) | 97.45±7.87 |
| SBP (mmHg) | 128.60±17.13 |
| DBP (mmHg) | 74.79±10.32 |
| MAP (mmHg) | 92.73±11.18 |
| VFA (cm^2^) | 92.28±38.06 |
| SFA (cm^2^) | 177.00(139.00,215.40) |
| **Metabolic Parameters** |  |
| Fasting plasma glucose (mmol/L) | 9.88(7.73,12.65) |
| Fasting plasma insulin (µIU/mL) | 7.48(4.40,10.83) |
| Fasting C-peptide (ng/mL) | 2.43±1.09 |
| 2h plasma glucose (mmol/L) | 19.19±5.22 |
| 2h plasma insulin (µIU/mL) | 31.25(16.24,44.62) |
| 2h C-peptide (ng/mL) | 4.49(3.24,6.73) |
| HbA1c (%) | 9.50(8.00,11.00) |
| HOMA-IR | 3.29(1.92,4.66) |
| HOMA-ISI | 0.37(0.22,0.56) |
| ALT (U/L) | 21.20(14.60,34.90) |
| AST (U/L) | 17.50(13.50,24.70) |
| ALP (U/L) | 71.00(58.00,86.00) |
| γ-GGT (U/L) | 28.00(20.00,47.00) |
| Albumin (g/L) | 40.40(38.20,42.50) |
| Blood urea nitrogen (mmol/L) | 5.24(4.38,6.41) |
| Creatinine (μmol/L) | 59.70(49.70,69.70) |
| Uric acid (μmol/L) | 286.00(230.00,342.00) |
| TCHOL (mmol/L) | 4.90±1.17 |
| TG (mmol/L) | 1.94(1.38,2.89) |
| HDL-c (mmol/L) | 1.06(0.89,1.26) |
| LDL-c (mmol/L) | 2.82±0.92 |

Abbreviations: CAD: coronary artery disease; BMI: body mass index; NC: neck circumference; WC: waist circumference; HC: hip circumference; SBP: systolic blood pressure; DBP: diastolic blood pressure; MAP: mean arterial pressure; VFA: visceral fat area; SFA: subcutaneous fat area；HOMA-IR: homeostasis model assessment of insulin resistance; HOMA-ISI: homeostasis model assessment of insulin sensitivity index; ALT: alanine aminotransferase; AST: aspartate aminotransferase; ALP: alkaline phosphatase; γ-GGT: gamma-glutamyl transferase; TCHOL: total cholesterol; TG: triglyceride; HDL-c: high-density lipoprotein cholesterol; LDL-c: low-density lipoprotein cholesterol.
