# Supplementary material for: Monocyte to High-Density Lipoprotein Cholesterol Ratio at the Nexus of Type 2 Diabetes Mellitus Patients With Metabolic-Associated Fatty Liver Disease
Source: Front Physiol. 2021 Dec 17;12:762242. doi: 10.3389/fphys.2021.762242 (PMC8718696; doi:10.3389/fphys.2021.762242)
Supplement: Supplementary file 2 [file Table_2.docx]

| Supplementary Table 2. The clinical and biochemical characteristics of different group according to the value of cutoff | | | |
| --- | --- | --- | --- |
| Variables | >cutoff point (0.388, n= 593) | < cutoff point (0.388, n= 458) | *P* value |
| **Age (years)** | **55.00(45.00, 63.00)** | **58.00(52.00, 66.00)** | **<0.001** |
| **Male (n,%)** | **427(72.01)** | **216(47.16)** | **<0.001** |
| **Smoking (n,%)** | **189(31.87)** | **89(19.43)** | **<0.001** |
| Alcohol intake (n,%) | 89(15.01) | 50(10.92) | 0.052 |
| Hypertension (n,%) | 324(54.64) | 247(53.93) | 0.819 |
| History of CAD (n,%) | 44(7.42) | 38(8.30) | 0.599 |
| **Dyslipidemia (n,%)** | **528(89.04)** | **314(68.56)** | **<0.001** |
| **Antidiabetic drug (n,%)** | **466(78.58)** | **386(84.28)** | **0.019** |
| **Height** **(cm)** | **167.84±8.24** | **163.81±8.38** | **<0.001** |
| **Weight** **(kg)** | **72.60±12.60** | **65.87±10.92** | **<0.001** |
| **BMI** **(kg/m^2^)** | **25.70±3.46** | **24.50±3.20** | **<0.001** |
| **NC** **(cm)** | **39.56±5.20** | **37.38±3.79** | **<0.001** |
| **WC (cm)** | **93.11±9.77** | **89.17±9.03** | **<0.001** |
| **HC (cm)** | **98.00(94.00,103.00)** | **96.00(92.00,100.00)** | **<0.001** |
| SBP (mmHg) | 128.78±17.08 | 128.37±17.21 | 0.839 |
| **DBP (mmHg)** | **75.30±9.96** | **74.13±10.75** | **0.036** |
| MAP (mmHg) | 93.13±10.95 | 92.21±11.47 | 0.186 |
| **VFA** **(cm^2^)** | **99.22±38.24** | **83.29±35.92** | **<0.001** |
| **SFA (cm^2^)** | **179.10(143.05,224.00)** | **169.75(134.00,204.10)** | **<0.001** |
| Fasting plasma glucose (mmol/L) | 10.43±3.28 | 10.12±3.35 | 0.111 |
| **Fasting plasma insulin (µIU/mL)** | **8.26(4.72,11.52)** | **6.81(3.93,10.66)** | **<0.001** |
| **Fasting C-peptide (ng/mL)** | **2.60±1.07** | **2.21±1.07** | **<0.001** |
| 2h plasma glucose (mmol/L) | 19.34±5.06 | 18.99±5.41 | 0.187 |
| **2h** **plasma insulin (µIU/mL)** | **32.60(17.51,46.81)** | **29.83(15.17,41.10)** | **0.024** |
| **2h C-peptide (ng/mL)** | **4.69(3.34,6.88)** | **4.31(3.04,6.57)** | **0.008** |
| HbA1c (%) | 9.64±2.16 | 9.51±2.21 | 0.261 |
| **HOMA-IR** | **3.64(2.20, 5.26)** | **2.82(1.68, 4.56)** | **<0.001** |
| **HOMA-ISI** | **0.33(0.19, 0.48)** | **0.39(0.24,0.60)** | **<0.001** |
| **ALT** **(U/L)** | **22.60(15.70, 40.05)** | **19.45(13.00,30.00)** | **<0.001** |
| AST (U/L) | 18.00(13.50, 25.00) | 17.00(13.50,24.00) | 0.334 |
| ALP (U/L) | 71.00(57.00, 86.00) | 71.00(58.00, 87.00) | 0.486 |
| **γ-GGT (U/L)** | **32.00(22.00, 51.00)** | **26.00(17.00, 41.00)** | **<0.001** |
| Albumin (g/L) | 40.30(38.10, 42.35) | 40.50(38.40,42.50) | 0.233 |
| Blood urea nitrogen (mmol/L) | 5.29(4.39, 6.41) | 5.21(4.36,6.39) | 0.675 |
| **Creatinine (μmol/L)** | **61.00(51.60,70.75)** | **57.65(47.98,67.83)** | **0.003** |
| **Uric acid (μmol/L)** | **300.00(243.50,352.00)** | **268.50(214.00,322.25)** | **<0.001** |
| **TCHOL** **(mmol/L)** | **4.76±1.10** | **5.09±1.23** | **<0.001** |
| **TG (mmol/L)** | **2.19(1.55, 3.20)** | **1.64(1.17, 2.47)** | **<0.001** |
| **HDL-c (mmol/L)** | **0.96±0.23** | **1.30±0.33** | **<0.001** |
| **LDL-c (mmol/L)** | **2.73±0.89** | **2.93±0.95** | **0.002** |
| **WBC** **(∗10^9^ /L)** | **6.40(5.50, 7.70)** | **5.40(4.50, 6.20)** | **<0.001** |
| **Neutrophil** **(∗10^9^ /L)** | **3.60(2.90, 4.60)** | **3.00(2.40, 3.80)** | **<0.001** |
| **Monocyte (∗10^9^ /L)** | **0.50(0.40,0.60)** | **0.40(0.30,0.40)** | **<0.001** |
| **Lymphocyte (∗10^9^ /L)** | **2.14±0.77** | **1.80±0.57** | **<0.001** |
| **Platelet (∗10^9^ /L)** | **200.04±56.63** | **186.12±52.84** | **<0.001** |
| **NHR** | **3.85(3.01, 5.06)** | **2.39(1.78, 3.11)** | **<0.001** |
| NLR | 1.72(1.35, 2.32) | 1.69(1.28, 2.31) | 0.289 |
| **PLR** | **94.76(73.98,120.00)** | **104.60(81.59, 133.39)** | **<0.001** |
| **MAFLD (n,%)** | **460(77.57)** | **285(62.22)** | **<0.001** |

Abbreviations: CAD: coronary artery disease; BMI: body mass index; NC: neck circumference; WC: waist circumference; HC: hip circumference; SBP: systolic blood pressure; DBP: diastolic blood pressure; MAP: mean arterial pressure; VFA: visceral fat area; SFA: subcutaneous fat area; HOMA-IR: homeostasis model assessment of insulin resistance; HOMA-ISI: homeostasis model assessment of insulin sensitivity index; ALT: alanine aminotransferase; AST: aspartate aminotransferase; ALP: alkaline phosphatase; γ-GGT: gamma-glutamyl transferase; TCHOL: total cholesterol; TG: triglyceride; HDL-c: high-density lipoprotein cholesterol; LDL-c: low-density lipoprotein cholesterol; WBC: white blood cell; MHR: monocyte/HDL-c; NHR: neutrophil/HDL-c; NLR: neutrophil/lymphocyte; PLR: platelet/lymphocyte; MAFLD: metabolic‑associated fatty liver disease.
